# Chemical Conjugation of Iron Oxide Nanoparticles for the Development of Magnetically Directable Silk Particles

**DOI:** 10.1021/acsami.4c17536

**Published:** 2025-02-03

**Authors:** Ande X. Marini, Golnaz N. Tomaraei, Justin S. Weinbaum, Mostafa Bedewy, David A. Vorp

**Affiliations:** † Department of Bioengineering, University of Pittsburgh, Pittsburgh, Pennsylvania 15261, United States; ‡ Department of Industrial Engineering, University of Pittsburgh, Pittsburgh, Pennsylvania 15261, United States; § McGowan Institute for Regenerative Medicine, University of Pittsburgh, Pittsburgh, Pennsylvania 15219, United States; ∥ Department of Pathology, University of Pittsburgh, Pittsburgh, Pennsylvania 15261, United States; ⊥ Department of Mechanical Engineering and Materials Science, University of Pittsburgh, Pittsburgh, Pennsylvania 15261, United States; 6 Department of Chemical and Petroleum Engineering, University of Pittsburgh, Pittsburgh, Pennsylvania 15261, United States; 7 Department of Surgery, University of Pittsburgh, Pittsburgh, Pennsylvania 15213, United States; 8 Department of Cardiothoracic Surgery, University of Pittsburgh, Pittsburgh, Pennsylvania 15213, United States; 9 Clinical & Translational Sciences Institute, University of Pittsburgh, Pittsburgh, Pennsylvania 15261, United States; 10 Magee Women’s Research Institute, University of Pittsburgh, Pittsburgh, Pennsylvania 15213, United States

**Keywords:** Silk microparticles, iron oxide nanoparticles, magnetic targeting, materials characterization, regenerated silk fibroin, magnetic guidance

## Abstract

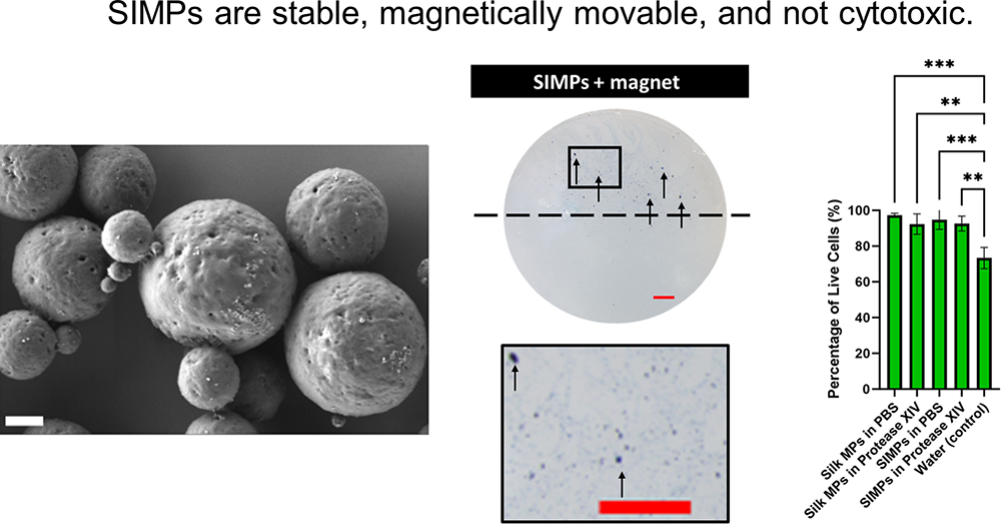

Magnetically directable materials containing iron oxide nanoparticles (IONPs) have been utilized for a variety of medical applications, including localized drug delivery. Regenerated silk fibroin (RSF) has also been used in numerous regenerative medicine and drug delivery applications, given its biocompatibility and tunable properties. In this work, we explored the hypothesis that chemically conjugating IONPs to RSF to anchor the IONPs to silk microparticles would provide better magnetic guidance than nonconjugated IONPs untethered to silk microparticles. IONPs were fabricated using a coprecipitation method and conjugated with glutathione (GSH) prior to mixing with RSF. IONPs incorporated into RSF were mixed with potassium phosphate buffer to fabricate microparticles. IONPs with and without GSH were characterized for particle size, shape, morphology, GSH conjugation efficiency, and composition. Silk iron microparticles (SIMPs) were also characterized for particle size, shape, and composition and tested for stability, degradation properties, magnetic movability, and cytotoxicity. IONPs demonstrated a uniform size distribution and spherical morphology. Conjugation of IONPs with GSH was verified through changes in the X-ray photoelectron spectroscopy (XPS) and Fourier-transform infrared spectroscopy (FTIR) spectra. IONPs and RSF were able to be chemically conjugated and fabricated into SIMPs, which demonstrated a spherical and porous morphology. FTIR revealed an increased β-sheet content in SIMPs, suggesting that the IONPs may be inducing conformational changes in the silk fibroin. SIMPs showed stability up to 4 weeks in ultrapure water and rapid enzymatic degradation within 24 h. SIMPs were able to be moved magnetically through solution and through a hydrogel and were not cytotoxic.

## Introduction

Magnetic materials have been used for a variety of medical applications, including drug targeting and delivery.^1−5^ Magnetic movement of materials may provide a minimally- or even noninvasive method for treatment of disease, as an external magnetic field can be used to move the desired magnetic material to the region of interest.^6^ These magnetic materials can either be directly labeled with a drug of choice^7^ or incorporated into a carrier.^8^ Iron oxide nanoparticles (IONPs) have shown utility for a variety of these applications^1,9^ and are noncytotoxic^5,10^ and biocompatible depending on their size, coating, and dosage. Typically, they can be cleared and degraded by the reticuloendothelial system,^11^ but they may also be secreted through the renal system via modification with polymer coatings.^12^ These characteristics make IONPs excellent candidates for magnetizing substances for drug delivery and regenerative medicine applications.^13,14^

*Bombyx mori* silkworm cocoons have been the most common source of silk fibroin used for a variety of biological applications, some of which have been FDA approved.^15,16^ From hydrogels^17^ to particles,^18,19^ the versatility of silk fibroin is vast. Silk fibroin is both degradable^20−22^ and biocompatible, only eliciting a low immune response. It also has tunable material characteristics based on how it is fabricated. Given its biocompatibility, silk has been used as a platform for drug delivery.^23,24^

Silk fibroin is also commonly processed for biomedical and tissue engineering applications into diverse formats^25^ including fibers,^26^ films,^27^ particles,^28^ hydrogels,^29,30^ aerogels,^31^ and 3D porous scaffolds.^20,32,33^ Previously, our lab has reported success in the use of silk fibroin to generate tissue engineered vascular grafts.^20,32,33^ Inorganic nanoparticles, including IONPs, have been added to silk fibroin particles to impart magnetic responsiveness to enable their precise manipulation and tracking.^34,35^

While IONPs are typically directly incorporated into silk fibroin material without specific functionalization or use of chemical bonding agents,^4,5,17,36−45^ magnetic nanoparticles coated with human serum albumin have been reported to enhance cell viability in magnetic silk fibroin scaffolds,^17^ indicating that magnetic nanoparticles could improve cellular activity. A common approach is physically blending IONPs into a regenerated silk fibroin (RSF) solution before processing the final material format.^5,17,37,38,40−43,45,46^ Importantly, most previous studies presume physical embedding of IONPs into the silk without direct characterization to confirm chemical bonding between the two, which we focus on in this work. In contrast, the primary focus in previous studies has been the characterization of composite material properties.

To address this gap, this study aimed to enhance the dispersion and retention of IONPs within silk microparticles (MPs) by utilizing glutathione (GSH). Thiol bond formation between IONPs and GSH was expected to facilitate interactions between IONPs and silk fibroin. We fabricated and characterized IONPs with or without a GSH tripeptide and then fabricated and evaluated conjugated silk-GSH-iron microparticles (SIMPs) and compared them with nonconjugated silk-iron microparticles (SIMPs-N). We demonstrate that the chemical conjugation between the IONPs, GSH, and RSF produces magnetically movable MPs that are suitable for potential biomedical applications due to their noncytotoxic nature.

## Methods

### Generation of Silk and Fabrication of IONPs

We followed a common method^47^ for RSF solution synthesis. Additional details can be found in the Supporting Information.

Magnetic IONPs were synthesized via the coprecipitation method, which involves the simultaneous precipitation of Fe^2+^ and Fe^3+^ ions in an alkaline solution. This process follows the reaction: Fe^2+^ + 2Fe^3+^ + 8OH^–^ → Fe_3_O_4_ + 4H_2_O. Precise control over parameters such as reactant concentration,^48^ temperature,^48,49^ stirring speed,^50^ and the removal of oxygen from the reaction environment^49^ is essential to govern the size, shape, morphology, and phase of the particles. Additional details can be found in the Supporting Information.

IONPs were conjugated with GSH (IONPs-GSH) following a scaled-up version of a procedure previously published.^6^ Additional details can be found in the Supporting Information.

### Incorporation of IONPs into an RSF Solution

As a tripeptide with a highly reactive thiol group, GSH effectively binds to heavy metal ions, forming metal-ion complexes that functionalize IONPs.^6,51,52^ The amine and carboxyl groups of GSH facilitate bonding with the carboxyl and amine functional groups on silk fibroin, enhancing interactions between nanoparticles and silk fibroin. To achieve a final RSF concentration of 50 mg/mL with or without IONPs, the original RSF solution was diluted with ultrapure water. Additional details can be found in the Supporting Information.

### Fabrication of Microparticles (MPs)

The silk solution (50 mg/mL) with or without iron (100 μL) was mixed with potassium phosphate buffer (2 M, pH = 8, 1000 μL) and incubated at 37 °C overnight to form MPs via a salting out method.^5^ Additional details can be found in the Supporting Information. MPs (Silk MPs, Silk-GSH-iron MPs (SIMPs), and Silk-iron MPs (SIMPs-N)) were stored at 4 °C until ready for use.

### IONP Characterization

Both IONPs-GSH and IONPs were characterized using SEM to assess particle size, shape, and morphology variation; thermal gravimetric analysis (TGA) to evaluate GSH conjugation efficiency; X-ray photoelectron spectroscopy (XPS) to confirm Fe_3_O_4_ synthesis and examine the elemental composition and chemical states of the nanoparticles; and Fourier-transform infrared spectroscopy (FTIR) to identify functional groups and chemical bonds. Additional details can be found in the Supporting Information.

### MP Materials Characterization

Three batches of Silk MPs and SIMPs suspensions in ultrapure water were analyzed shortly after preparation using SEM to examine the morphology and size, energy-dispersive X-ray spectroscopy (EDS) to determine elemental composition, and FTIR to evaluate variations in the secondary structure of the silk fibroin. Additional details can be found in the Supporting Information.

Silk MP and SIMP suspensions in ultrapure water were stored at 4 °C to assess stability over a period of 28 days. At specific time points, portions of each suspension were analyzed by SEM, EDS, and FTIR as described above. The results were compared to those obtained shortly after the preparation of the suspensions to evaluate the stability.

To assess the degradation of Silk MPs and SIMPs, samples were incubated in PBS or mixed 1:1 with Protease XIV (1 U/mL, P5147, Millipore Sigma, St. Louis, MO) at 37 °C for 1, 4, or 7 days. To minimize additional degradation from residual protease, the resuspended samples were promptly cast onto Si wafer chips for SEM, EDS, and FTIR analysis, as described previously. Additional details can be found in the Supporting Information.

### Dynamic Light Scattering and Coulter Counter Analysis

Silk MPs and SIMPs were resuspended in ultrapure water and analyzed for zeta potential and size using dynamic light scattering (DLS) and Coulter Counter analysis, respectively. For DLS, a Nano-ZS90 Zetasizer (Malvern Panalytical, Westborough, MA) was used. For measuring the zeta potential, 1 mL of resuspended MPs was added to a capillary zeta cell (Malvern Panalytical, DTS1070) and allowed to equilibrate to 25 °C for 120 s. Particles were measured using an automated program with three runs for the measurements. The refractive index of the particles was assumed to be 1.5605. For the coulter counter to measure size distribution, a Multisizer-3 Coulter Counter (Beckman Coulter, Brea, CA) was used. A volume of 50 μL of MPs was mixed with approximately 10 mL of isotone buffer according to the manufacturer’s protocols. If particle counts were too low, additional MPs were added to up to 300 μL.

### Magnetic Movability Analysis

**Figure 1:**
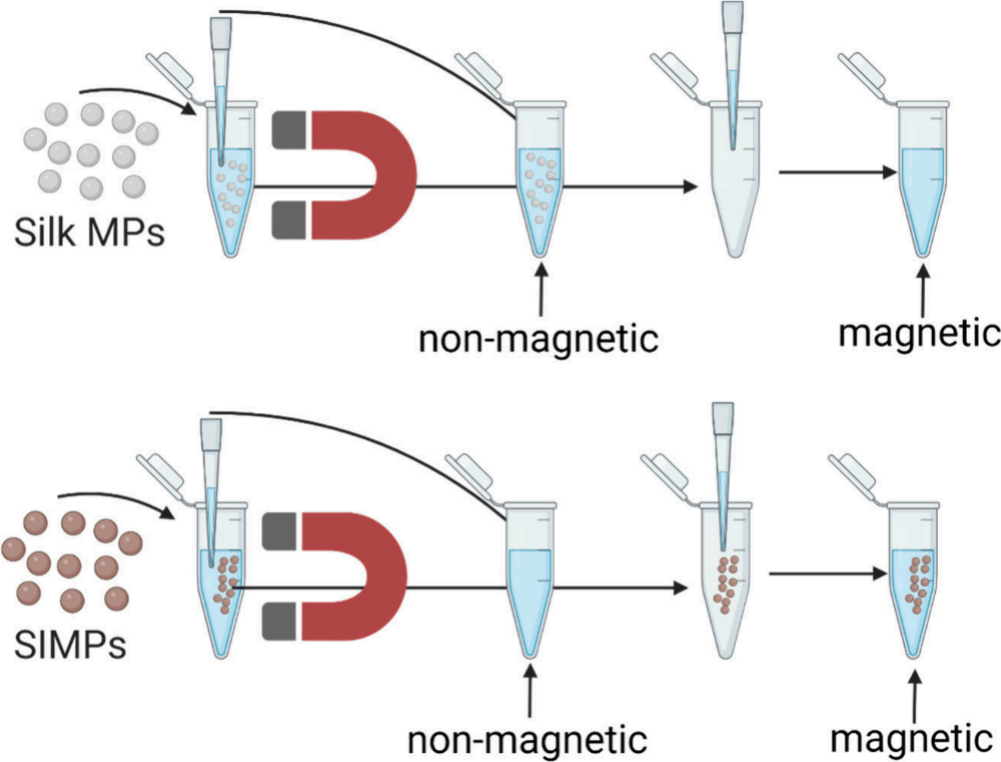
**Visual representation of a separation assay of Silk MPs and SIMPs with a magnet.** Each fraction (magnetic and non-magnetic) was used in a Lowry assay to detect the concentration of protein. These fractions were also characterized by SEM to see individual MPs. The image was made on Biorender.com.

Three different batches of RSF and three different batches of IONPs-GSH were mixed together to create the silk-iron solution. This solution was used to generate three batches of SIMPs at varying IONPs-GSH concentrations (0–50 in 10 mg/mL increments) to determine the optimal concentration of IONPs-GSH to magnetically move the SIMPs. The optimal concentration was determined with a separation assay followed by a Lowry assay (23240, ThermoFisher Scientific, Pittsburgh, PA) to measure total protein in the samples (i.e., silk). As outlined in Figure 1, a 100 μL aliquot of MPs was taken to be used for separation assays. By physically separating the particles into their magnetic and non-magnetic fractions, we were able to quantify the concentration of protein moved magnetically and visually see the moved particles with SEM. A Lowry assay (23240, ThermoFisher Scientific) was performed on the samples to determine protein concentration as described in the manufacturer’s protocol. More details can be found in the Supporting Information.

Additionally, fibrin gels were made as previously described,^53,54^ replacing the cellular component with Silk MPs or SIMPs; 200 μL of solution was used per gel. Gels exposed to the magnet were exposed throughout the entire gelation process. Following gelation, fibrin gels were fixed and stained with Prussian Blue stain to identify iron and imaged. Additional details can be found in the Supporting Information.

### Interference Analysis of GSH, IONPs, and IONPs-GSH on Protein Concentration Readings

Detection of possible interference from GSH, IONPs, and IONPs-GSH by the Lowry assay was performed. An equal ratio of the interfering agent to BSA protein was determined based on the concentration of GSH or IONPs-GSH to the concentration of silk, assuming 25 mg/mL IONPs-GSH per 50 mg/mL silk, 13 mg of GSH per 16 mg of IONP, and 1 mg of IONP to 1 mg of IONP-GSH. The standard curve containing potential interfering agents was then analyzed and compared to the actual standard curve using a Lowry assay (23240, Thermofisher) as described previously. Additional methods of interference of IONPs-GSH can be found in the Supporting Information.

### Cytotoxicity of MPs

Human aortic vascular smooth muscle cells (SMCs) from Cell Applications (354–05a, San Diego, CA) were cultured at 37 °C and 5% CO_2_ (3530, Isotemp, ThermoFisher Scientific) in supplemented basal media (311 K-500, Cell Applications Inc.) between passages 6–10. SMCs were plated at a density of 40,000 cells/well in 1 mL of supplemented basal media in 24 well plates. Cells were cultured overnight to adhere to the sample prior to treatment. Transwells (0.4 μm pores, 662641, Greiner, Monroe, NC) were placed on top, and 150 μL of treatment was added to each transwell. Treatments consisted of Silk MPs or SIMPs resuspended in either PBS or Protease XIV (0.01 U/mL). Cells were incubated with treatment for 24 h. Cytotoxicity was determined using a LIVE/DEAD stain (R37601, (488/570) Invitrogen, Carlsbad, CA). Cells were imaged with a Nikon Ti2 wide lens fluorescent microscope (Nikon, Melville, NY) with living cells stained green (488 nm) and dead cells stained red (570 nm). Additional details can be found in the Supporting Information.

### Statistical Analysis

Statistical analyses were performed to determine significance between groups, with *p* < 0.05 used to represent significance. A one-way analysis of variance (ANOVA) with a posthoc Tukey’s test was used to analyze differences in protein concentration prior to separation assays across three different SIMP batches and across different IONPs-GSH concentrations. An ANOVA with a posthoc Dunnett’s test was used to determine differences between the ratio of protein in magnetic and non-magnetic fractions and differences in the number of dead cells following Silk MP and SIMP treatment. Results are reported as mean ± standard deviation (S.D.). All results were analyzed in GraphPad Prism 9 (GraphPad, San Diego, CA).

## Results and Discussion

### Characterization of IONPs

**Figure 2:**
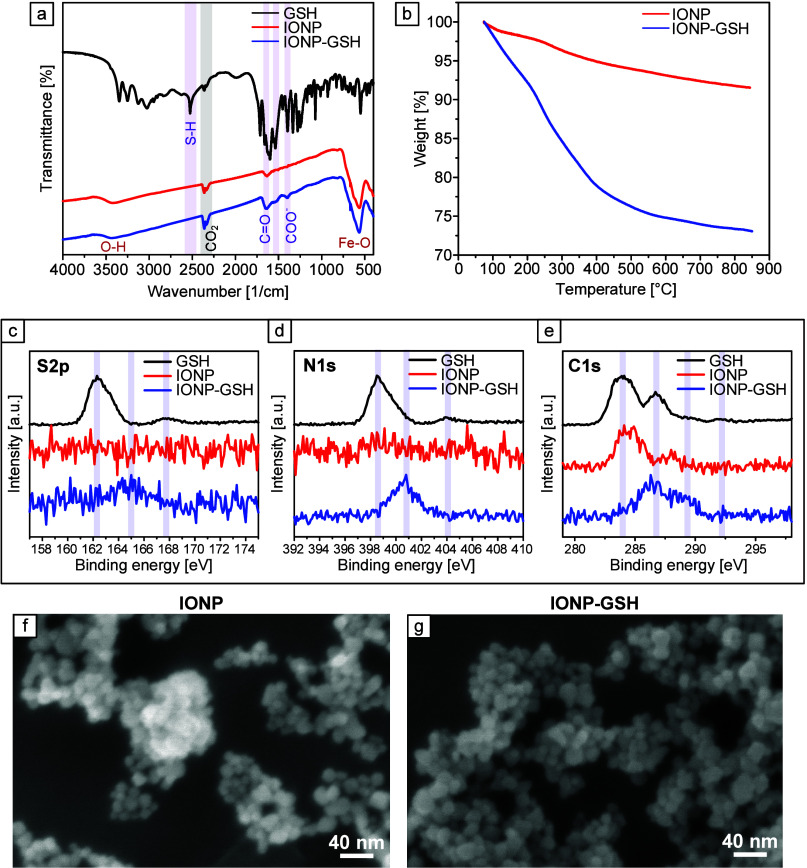
**Comparative characterization of IONP and IONP-GSH.** a) FTIR spectra of GSH, IONP, and IONP-GSH. (b) TGA analysis reveals a higher mass loss in IONP-GSH. (c) XPS S 2p, (d) XPS N 1s, and (e) XPS C 1s spectra of GSH, IONP, and IONP-GSH together confirm conjugation. (f, g) SEM images depicting uniform particle size and shape in both IONP and IONP-GSH, with slightly enhanced nanoparticle dispersion in IONP-GSH.

Figure 2 illustrates the characterization of IONPs and IONPs-GSH. The FTIR spectra (Figure 2a) reveal a peak at 564 cm^–1^ in both samples, indicating an Fe–O bond. The IONPs-GSH spectrum shows additional peaks at 1400 cm^–1^ (symmetric stretching vibration of COO^–^) and 1520 cm^–1^ (amide bond for GSH) and an enhanced peak at 1640 cm^–1^. The absence of the S–H bond peak at 2524 cm^–1^ in IONPs-GSH further confirms the conjugation. The peak at around 2360 cm^–1^ in all three samples corresponds to CO_2_. The broad peak spanning 3000–3600 cm^–1^ in both IONP and IONPs-GSH arises from the O–H stretching vibrations. TGA analysis in Figure 2b shows higher mass loss in IONPs-GSH, indicating GSH coverage. The XPS S 2p spectrum (Figure 2c) shows a peak at 165 eV in IONPs-GSH, suggesting thiol bond formation, which is absent in the IONP spectrum. The XPS N 1s spectrum (Figure 2d) shows peaks at 398.5 and 404 eV in GSH, and at 400.8 eV in IONPs-GSH, confirming changes in the nitrogen environment upon conjugation. The XPS C 1s spectrum (Figure 2e) shows peaks at 286.8 and 289.3 eV in IONPs-GSH, indicating specific carbon functional groups from GSH. SEM images (Figures 2f and 2g) reveal uniform particle shapes and sizes, with improved dispersion in IONPs-GSH.

The deconvoluted XPS Fe 2p spectrum of IONPs (Figure S1a) shows peaks at 709.7 and 722.9 eV, corresponding to the 2p3/2 and 2p1/2 states of Fe^2+^, respectively, and peaks at 711.3 and 724.9 eV, corresponding to the 2p3/2 and 2p1/2 states of Fe^3+^, respectively. Satellite peaks are observed at 717.7 and 731.1 eV. These results align with previous reports for Fe_3_O_4_.^55,56^ Considering the Fe^3+^:Fe^2+^ ratio of 2:1 in Fe_3_O_4_, the areas of Fe^3+^ peaks were constrained to be twice those of corresponding Fe^2+^ peaks during deconvolution. Peak position, full width at half-maximum (fwhm), the percentage of Gaussian–Lorentzian mix, and the areas of Fe^2+^ and satellite peaks were used as fitting parameters, resulting in a satisfactory fit (Figure S1a) with parameters listed in Table S1. The deconvoluted O 1s spectrum (Figure S1b) shows peaks at 529.5 eV, corresponding to the O^2–^ ions in the Fe_3_O_4_ lattice, and at 531.1 eV, likely due to C=O bonds from surface contamination. Surface hydroxyl groups from adsorbed moisture may also contribute to peaks in this region.^55^

### Characterization of MPs

**Figure 3:**
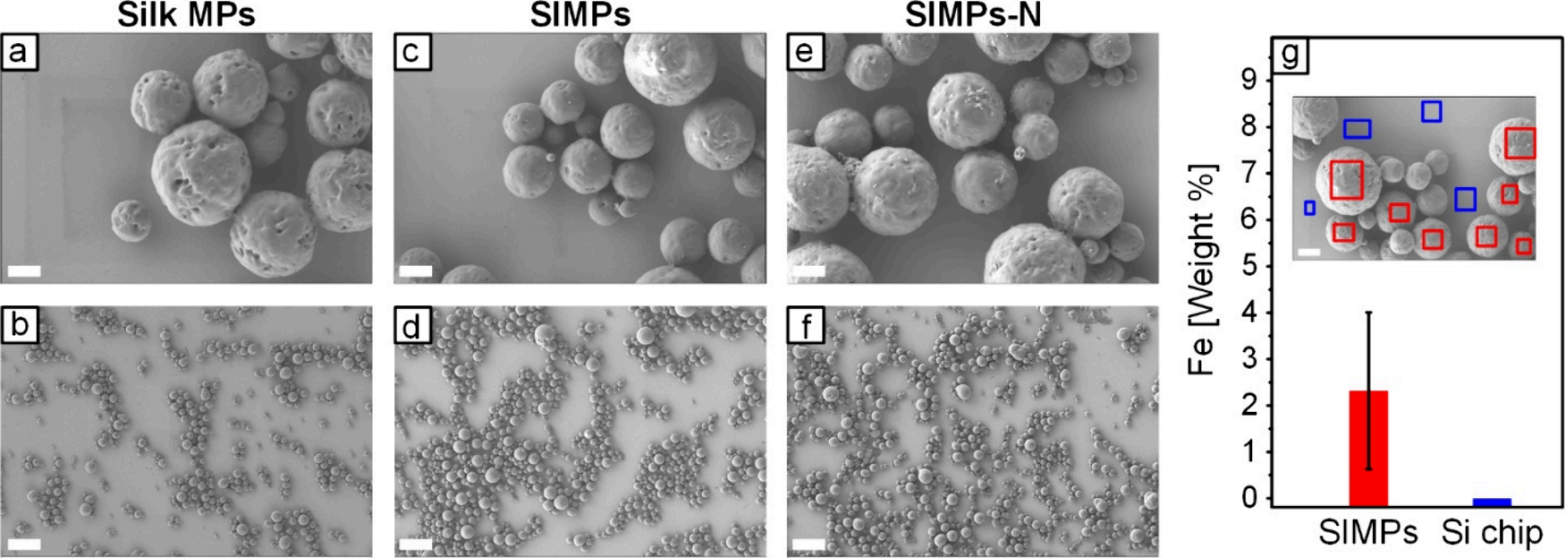
**SEM images of MPs prepared using the salting out method.** Representative (a) high magnification and (b) low magnification images show Silk MPs without IONPs. Representative (c) high and (d) low magnification images depict SIMPs with IONPs conjugated with GSH. Representative (e) high and (f) low magnification images show SIMPs-N with IONPs that are not conjugated with GSH. (g) SEM image with overlaid rectangles indicating the areas where the EDS spectra were gathered. The bar chart presents the average weight percent of Fe measured in these spectra for SIMPs and Si chip regions. The average weight percentage of Fe on the Si chip was consistently measured as zero, and thus, no error bars are shown for the blue column. Scale bars: Panels a, c, e, and g (1 μm) and panels b, d, and f (10 μm).

Representative SEM images at high and low magnifications for MPs without (Figures 3a and 3b) and with (Figure 3c-f) IONPs exhibited a spherical morphology with well-preserved structural integrity. The SIMPs had smoother surfaces with less porosity. Average particle sizes were 1.90 ± 0.84 μm, 1.63 ± 0.61 μm, and 1.40 ± 0.68 μm for Silk MPs, SIMPs, and SIMPs-N, respectively. The initial silk concentration is known to strongly influence MP size.^57−60^ Blue and red rectangles overlaid on the SEM image of Figure 3g indicate several EDS spectra that were collected from the Si chip and SIMPs, respectively. Si was detected but was excluded from elemental analysis. The average weight percent of iron from these spectra is presented as a bar chart in Figure 3g.

The EDS elemental maps of C, N, and O closely correspond to the particle morphology seen in the SEM images for both SIMPs-N (Figures S2a, S2f) and SIMPs (Figures S2g-S2l). However, the Fe map is less correlated to the particle morphology because of its lower concentration and weaker signal.

The amide I region of FTIR spectra of three batches (B1, B2, and B3) of each sample type—Silk MPs, SIMPs, and SIMPs-N—in Figure S3 demonstrate consistency across batches, highlighting reproducibility. All samples exhibit a peak around 1652 cm^–1^ (α-helix structure). The peaks at approximately 1624 and 1700 cm^–1^ (β-sheet secondary structure) are more pronounced in both SIMPs and SIMPs-N, especially in SIMPs. Peaks at about 1683 and 1695 cm^–1^ (β-turns) also intensify in both SIMPs and SIMPs-N. This enhancement of secondary structures may result from the IONP presence during microparticle formation. It could also be influenced by trace amounts of methanol or ethanol used during the final washing of the IONPs.

### Stability Analysis of MPs

**Figure 4:**
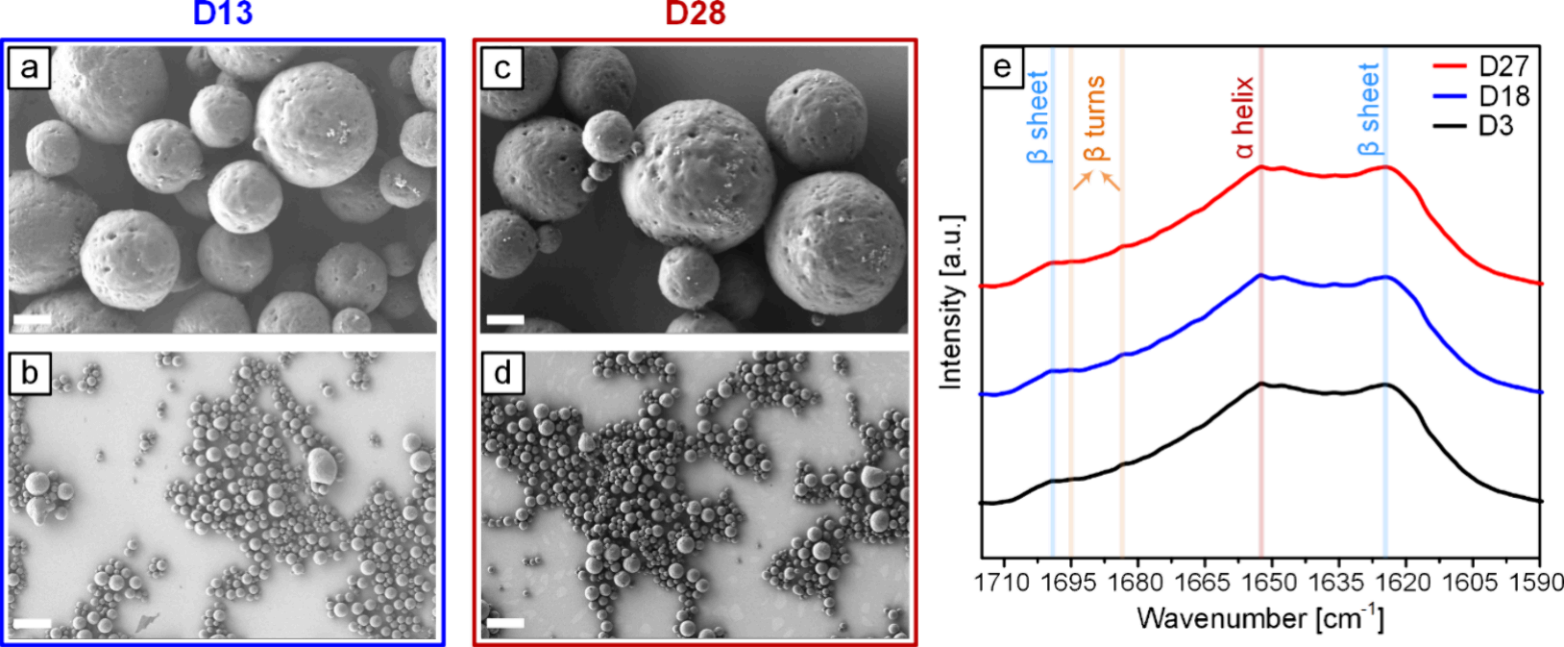
**Stability assessment of SIMPs stored at 4 °C in the refrigerator for 13 and 28 days.** SEM images at D13, at (a) high magnification and (b) low magnification, and at D28, at (c) high magnification and (d) low magnification, show that size and morphology remain unchanged over time. (e) Amide I FTIR spectra for one batch of SIMPs at D3, D18, and D27 show minimal changes, indicating a stable secondary structure. Scale bars: Panels a and c (1 μm) and panels b and d (10 μm).

Figure 4 shows SEM images of SIMPs at day 13 (D13) and day 28 (D28), as well as the amide I region of the FTIR spectra for one batch at D3, D18, and D27. Additionally, Figure S4 includes similar SEM images and FTIR spectra for Silk MPs and SIMPs-N. Comparing these SEM images with those in Figure 3 reveals that the particle size and morphology remain stable over time. The FTIR spectra also show minimal changes, further confirming the stability of the MPs in ultrapure water suspensions. Notably, the increased intensity of β-sheet and β-turn peaks in SIMPs and SIMPs-N, as described previously, persists even after prolonged exposure, suggesting the stability of their secondary structure throughout the exposure period.

### Degradation Analysis of MPs

**Figure 5:**
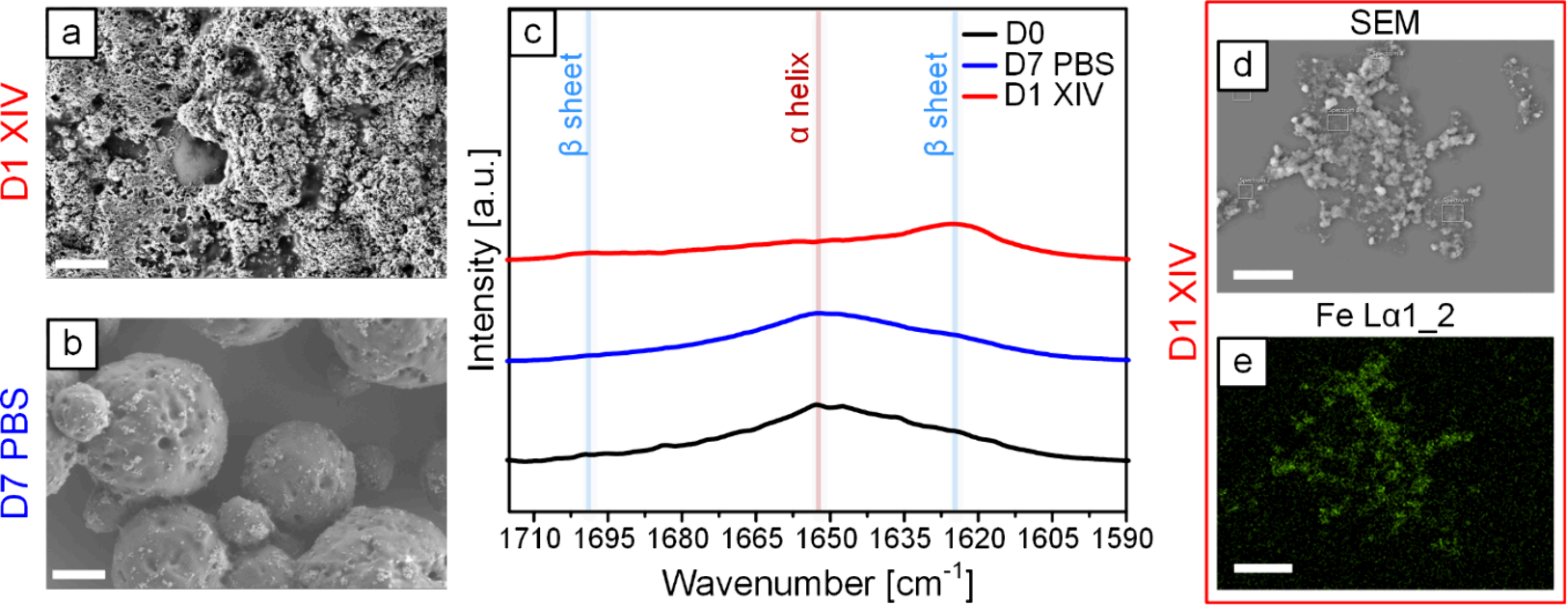
**Degradation analysis of SIMPs incubated in Protease XIV and PBS.** SEM image at (a) D1 of incubation in Protease XIV shows signs of degradation, while the SEM image at (b) D7 of incubation in PBS shows that SIMPs maintain structural integrity and spherical shape. (c) Amide I FTIR spectra for SIMPs without incubation (D0), at D1 in Protease XIV, and at D7 in PBS indicate minimal changes over 7 days in PBS, while 1 day in Protease XIV induces substantial β-sheet increase and α-helix reduction, consistent with SEM degradation observations. (d) Representative SEM image and (e) the corresponding Fe Kα1_2 EDS map of degraded SIMPs at D1 in Protease XIV support the localization of IONPS within the SIMPs. Scale bars: Panels a, b (1 μm) and panels d, e (10 μm).

SEM images of Silk MPs (Figure S5a,b) and SIMPs (Figures 5a and 5b) at D1 in Protease XIV and D7 in PBS show that both materials degrade in Protease XIV at D1 but retain their structural integrity and spherical shape in PBS at D7. Figure 5d shows a representative SEM image of degraded SIMPs at D1 in Protease XIV, and Figure 5e presents the corresponding EDS map for Fe Kα1_2, indicating the spatial distribution of iron within the analyzed area. The significant overlap between the SEM image and the EDS map confirms that IONPs were primarily localized within the SIMPs and remained associated with the degraded protein. Full EDS elemental maps are presented in Figure S6.

The amide I region of FTIR spectra at D1 in Protease XIV and D7 in PBS for both Silk MPs (Figure S5c) and SIMPs (Figure 5c) shows stability in PBS, consistent with the SEM images (Figures 5 and S5). However, at D1 in Protease XIV, both samples exhibited a substantial increase in the β-sheet peak (∼1624 cm^–1^) and a significant reduction in the α-helical peak (∼1652 cm^–1^). This spectral alteration can be attributed to the selective signal retention of stable structures, consistent with the accelerated degradation observed in the SEM images (Figures 5 and S5). The degradation analysis revealed the susceptibility of both Silk MPs and SIMPs to Protease XIV. As both SIMPs and Silk MPs were easily degraded in Protease XIV, they could release any potential cargo relatively easily in a degradative environment.

### DLS and Coulter Counter Analysis

**Figure 6:**
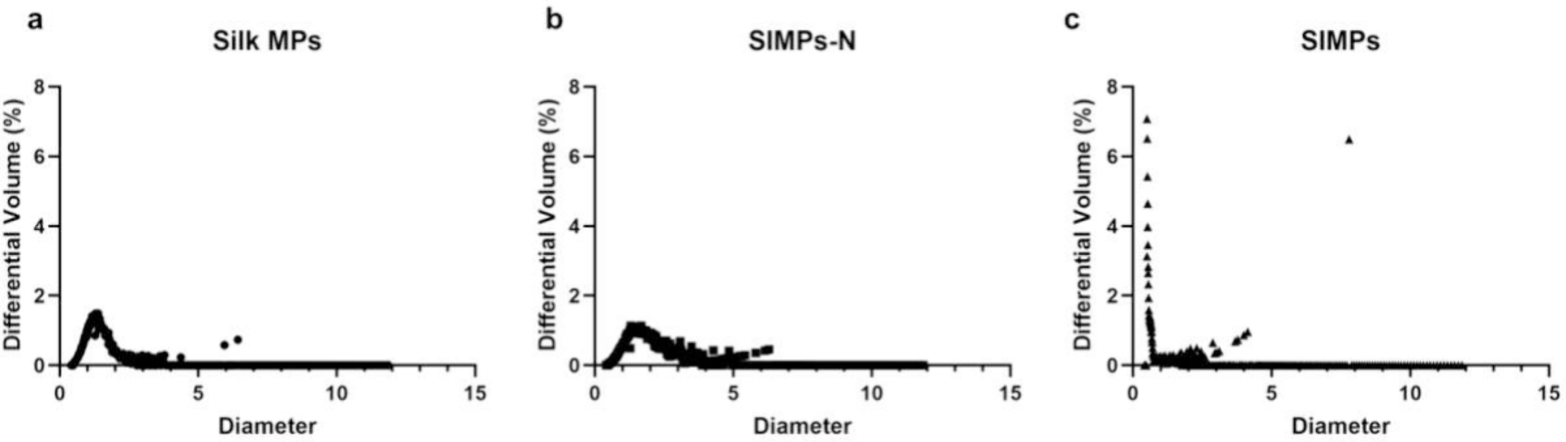
**Size distribution curves show similar diameters between Silk MPs and SIMPs-N but smaller diameters for SIMPs.** Three different types of microparticles (Silk MPs (a), SIMP-N (b), and SIMPs (c)) were analyzed for size. Silk MPs (1.43 ± 0.79 μm) and SIMPs-N (1.85 ± 1.02 μm) had similar diameters, while SIMPs had smaller diameters (0.57 ± 0.13 μm).

DLS revealed a negative zeta potential for all three different particle types (Silk MPs, SIMPs-N, and SIMPs). SIMPs-N had a more negative zeta potential (46.2 ± 0.4 mV) compared to the Silk MPs (−44.3 ± 0.6 mV, p = 0.0165) and SIMPs (−43.3 ± 0.7 mV, p = 0.0021); there were no significant differences between Silk MPs and SIMPs (p = 0.1677) (Table 1). CoulterCounter analysis showed that Silk MPs (1.43 ± 0.79 μm) and SIMPs-N (1.85 ± 1.02 μm) had similar diameters while SIMPs had smaller diameters overall (0.57 ± 0.13 μm) (Figure 6).

**Table 1:** Zeta Potential of Each Type of MP, with All Three Groups Demonstrating a Negative Zeta Potential^a^

parameter	value
Silk MPs	–44.3 ± 0.6 mV
SIMPs-N	–46.2 ± 0.4 mV
SIMPs	–43.3 ± 0.7 mV

a Three runs were performed (n = 3) to determine statistical differences between MP groups.

### Magnetic Movability Analysis

**Figure 7:**
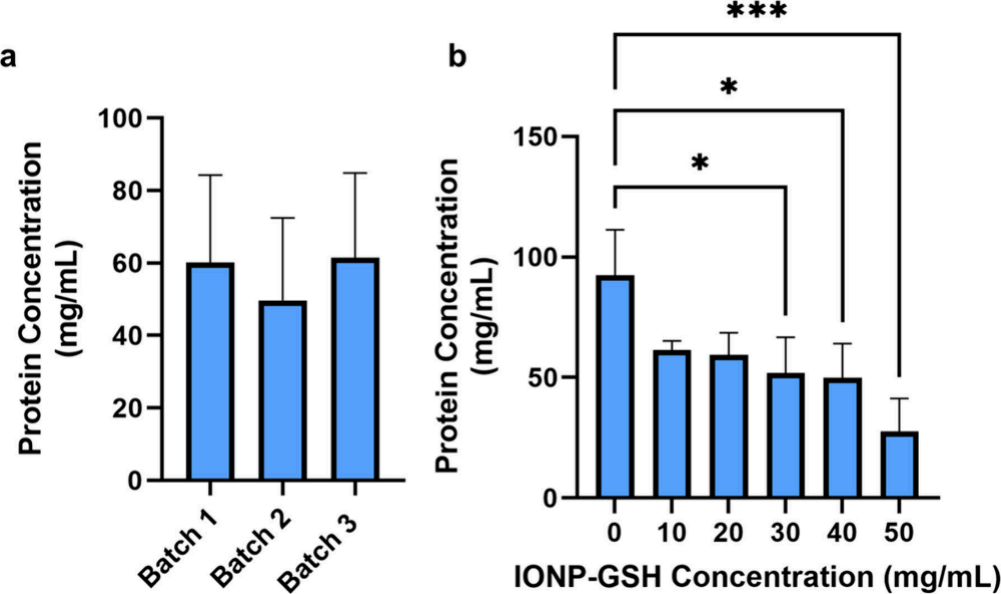
**Concentration of silk protein is relatively consistent across batches (a) but significantly different between iron concentrations (b)**. Three different batches of silk and IONPs-GSH were mixed at different concentrations of IONPs-GSH. Average concentration of protein across all three batches was not significantly different between batches (*p* = 0.726, 0.994, and 0.665). However, with iron concentrations higher than 20 mg/mL, there was a significant difference in protein concentration. * = *p* < 0.05, *** = *p* < 0.001.

**Figure 8:**
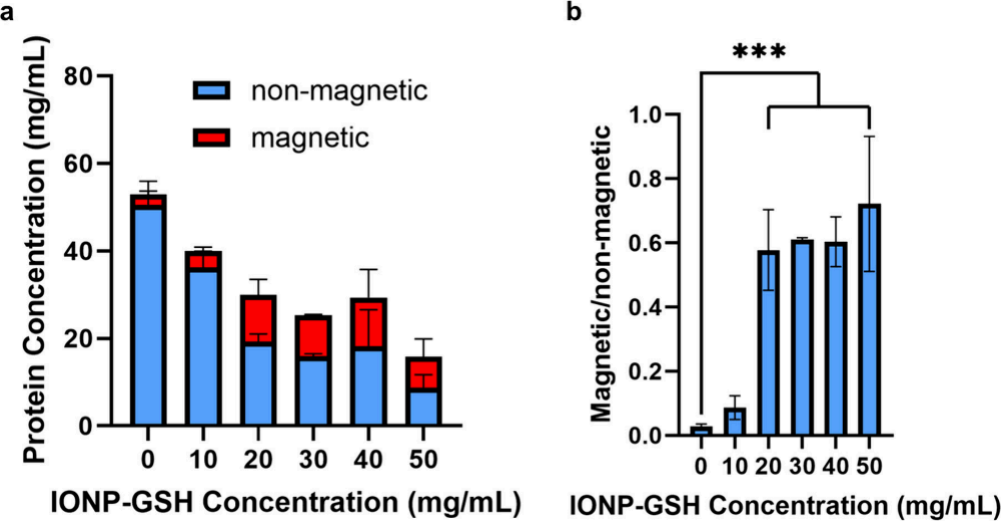
**Increasing IONP concentration increases magnetic movability of SIMPs.** The concentration of protein detected in the magnetic fraction portion of the SIMPs increased at 20 mg/mL IONPs and plateaued afterward (a). Comparing the ratio of protein in the magnetic fraction to the protein in the non-magnetic fraction showed this plateau effect (b). There was a significant difference in this ratio of protein between no IONPs (0 mg/mL) and 20 mg/mL and higher concentrations of IONPs. *** = *p* < 0.001.

Concentration of silk protein in SIMPs before separation was relatively consistent between the three batches (n = 6, 60.11 ± 24.15 mg/mL, 49.67 ± 22.79 mg/mL, and 61.48 ± 23.38 mg/mL, *p* > 0.05) with varying amounts of IONPs (Figure 7a and Table 2), indicating the reproducibility of our salting out technique. However, comparing the concentration of silk protein based on IONP-GSH concentration showed a difference between 0 and 30, 40, and 50 mg/mL (n = 3, 92.25 ± 19.08 mg/mL vs 51.98 ± 14.64 mg/mL, p = 0.0279, 49.91 ± 14.13 mg/mL, p = 0.0203, and 27.61 ± 13.72 mg/mL, p = 0.0007) (Figure 7b and Table 3). Comparing the concentration of protein between the separated magnetic and non-magnetic fractions of SIMPs showed that there were higher concentrations of protein in groups with higher concentrations of IONP-GSH (Figure 8a and Table 3). There was also a significant difference between the ratio of magnetic to non-magnetic fraction of groups with 0 mg/mL of IONP-GSH and concentrations of 20 mg/mL and above (Figure 8b and Table 3) (n = 3, 0.03 ± 0.01 vs 0.58 ± 0.136, p = 0.0002, 0.61 ± 0.015, p = 0.0001, 0.60 ± 0.08, p = 0.0001, and 0.72 ± 0.21, *p* < 0.0001); this threshold of 20 mg/mL could imply a limit of IONP incorporation within the silk.

**Table 2:** Comparison of Concentration of Protein (mg/mL) Detected by Lowry Assay between Three Batches across Six Different Iron Concentrations^a^

	Batch 1	Batch 2	Batch 3
Protein concentration (mg/mL)	60.11 ± 24.15	49.67 ± 22.79	61.48 ± 23.38

a (n = 6 per batch), p > 0.05 between all comparisons.

**Table 3:** Concentrations of Protein (mg/mL) Detected by Lowry Assay between Six Different Iron Concentrations across Three Different Batches, Concentrations of Protein between Non-magnetic and Magnetic Fractions, and Ratio of Protein in Each Fraction of Separated SIMPs (Magnetic/Non-magnetic)^a^

	parameter	value					
	Iron concentration (mg/mL)	0	10	20	30	40	50
	Protein concentration (mg/mL)	92.25 ± 19.08	61.35 ± 3.78	59.41 ± 9.11	51.98 ± 14.64	49.91 ± 14.13	27.61 ± 13.72
Fraction	Non-magnetic	50.59 ± 5.35	36.30 ± 3.70	19.33 ± 1.73	15.97 ± 0.53	18.25 ± 8.34	8.77 ± 2.95
	Magnetic	2.30 ± 0.83	3.70 ± 0.92	10.65 ± 3.54	9.32 ± 0.19	11.08 ± 6.45	7.09 ± 4.01
	Ratio	0.04 ± 0.01	0.09 ± 0.04	0.58 ± 0.13	0.61 ± 0.02	0.60 ± 0.08	0.72 ± 0.21

a (n = 3 per group).

**Figure 9:**
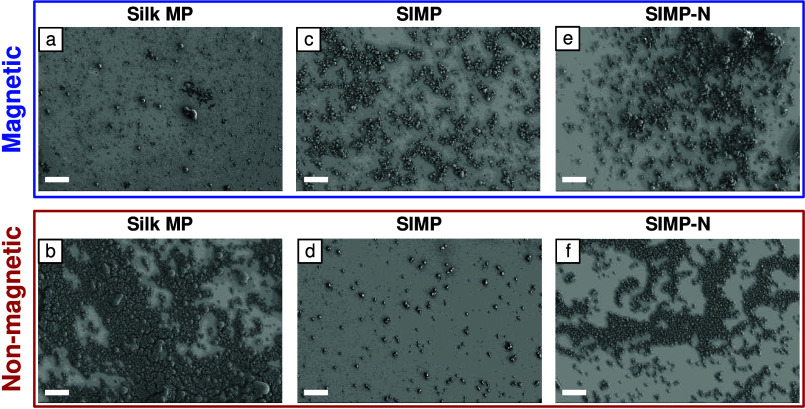
**Representative SEM images of (a, b) Silk MPs, (c, d) SIMPs, and (e, f) SIMPs-N, displaying their magnetic and non-magnetic separated fractions.** Unique particle distributions are observed: Silk MPs mostly in non-magnetic fraction, SIMPs predominantly in magnetic fraction, and SIMPs-N with a notably higher non-magnetic fraction count compared to SIMPs. The scale bars in all panels are 20 μm.

Representative SEM images of the magnetic and non-magnetic fractions of each sample type are shown in Figure 9. For Silk MPs (Figures 9a and 9b), most particles are in the non-magnetic fraction. In SIMPs, most particles are found in the magnetic fraction. In SIMPs-N, while fewer particles are in the non-magnetic fraction compared to Silk MPs, it is significantly higher than in the non-magnetic fraction of SIMPs. The more efficient magnetic movability of SIMPs is likely due to the enhanced incorporation of IONPs within the MPs through chemical conjugation.

**Figure 10:**
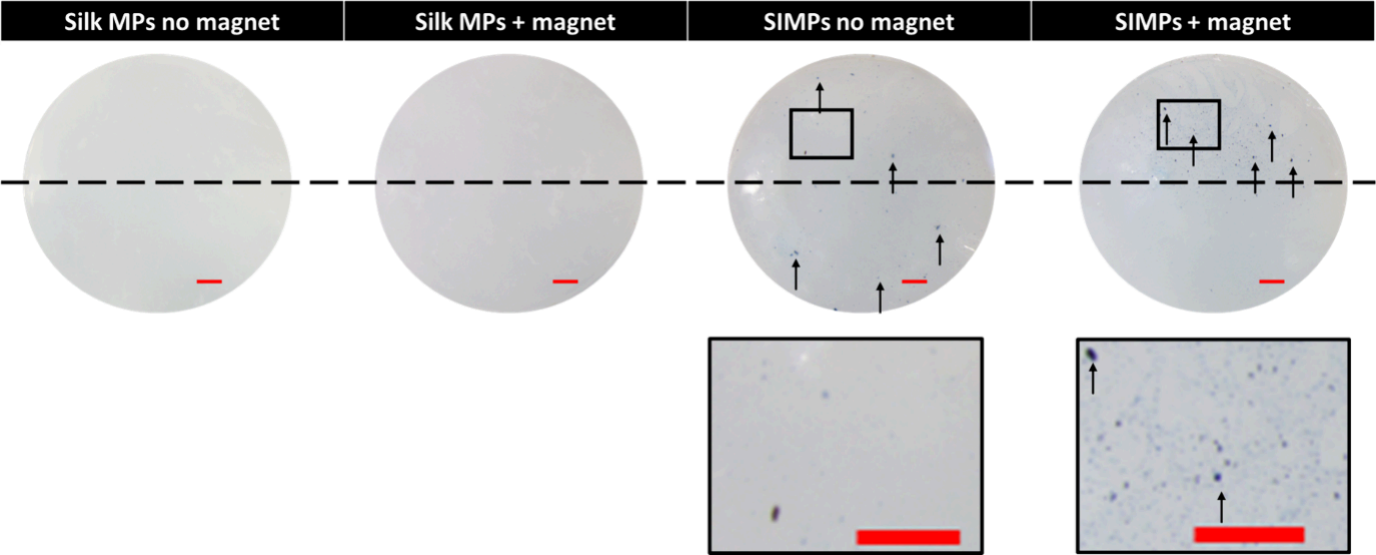
**SIMPs can be magnetically moved through a hydrogel.** Silk MPs and SIMPs were loaded into fibrin hydrogels and exposed to a neodymium magnet to see the magnetic movement of the particles. The neodymium magnet was placed at the top of the fibrin gels. Particles containing IONPs were able to be detected using a Prussian blue stain (indicated with black arrows), with higher concentrations of IONP containing particles (i.e., SIMPs) detected closer to the magnet (above the dashed line), indicating magnetic movability and detection. The black square shows a zoomed in image of the SIMPs with or without magnetic exposure, qualitatively showing a higher concentration of MPs in gels exposed to a magnet. Scale bar (red) = 1 mm.

SIMPs loaded into fibrin gels showed blue coloration after being stained with Prussian blue, a specific stain for iron, compared with Silk MPs, which were not stained blue. SIMPs exposed to the magnet appeared more concentrated as they were moved toward the magnet (Figure 10). Additional images of magnetic movement through the fibrin gel can be seen in the the Supporting Information (Figure S9).

### Interference of IONPs-GSH with Protein Concentration Measurements

Interference from the IONPs in the Lowry Protein Assay was detected and shown to increase as the concentration of IONPs in the SIMPs increased (Figure S7a). A relationship between the concentration of IONPs and the slope of all protein/BSA proteins was shown to be linear with an r^2^ value of 0.9979 (Figure S7b). Interference was not seen from GSH alone (Figure S8).

### Cytotoxicity of MPs

**Figure 11:**
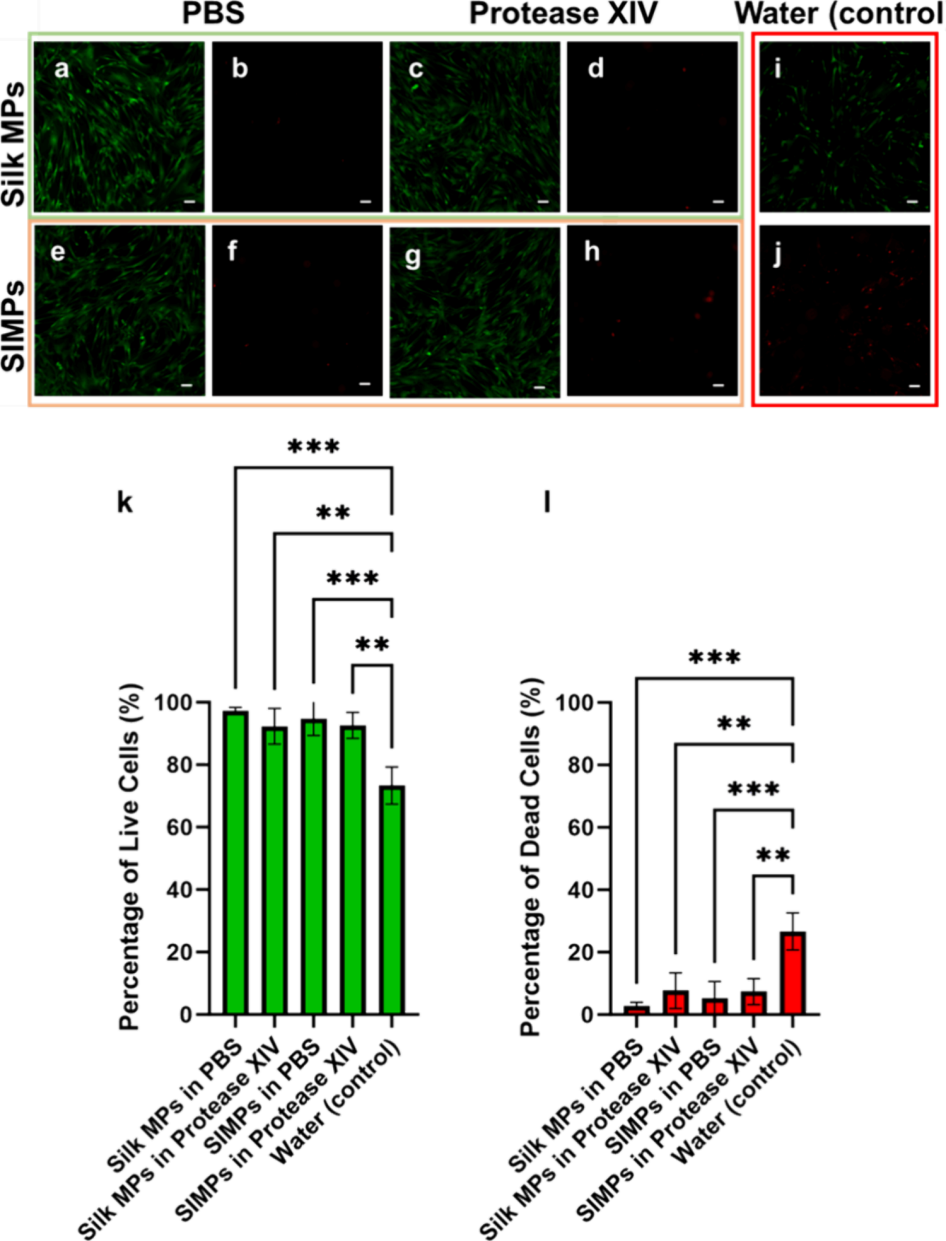
**LIVE/DEAD stain of SMCs cocultured with Silk MPs and SIMPs incubated in PBS or digested with Protease XIV showed low cytotoxicity of the particles.** SMCs were also cultured with water as a negative control. Green channels (a,c,e, and g) showed confluent live cells in all MP treated groups compared to the negative control (i). Red channels (b,d,f, and h) showed minimal amounts of red (dead) cells compared to the negative control (j). The number of live (green) and dead cells (red) was counted across treatments (*n* = 3), and the percentage of live and dead cells was compared to the negative control. There was a significantly higher percentage of live cells (k) and simultaneously a significantly lower percentage of dead cells (l) in the MP groups compared to the negative control. Scale bar = 100 μm ** = *p* < 0.01, *** = *p* < 0.001

Coculturing SIMPs and Silk MPs with SMCs did not induce cytotoxicity (Figure 11). All combinations of MPs and PBS or Protease XIV had lower cytotoxicity compared to the negative control (Silk MPs + PBS: n = 3, 97.2 ± 1.1% live cells, p = 0.0004, Silk MPs + Protease XIV: 92.3 ± 5.7% live cells, p = 0.0023, SIMPs + PBS: 94.7 ± 5.3% live cells, p = 0.0010, and SIMPs + Protease XIV: 92.6 ± 4.2% live cells, p = 0.0021 vs Water: 73.3 ± 5.9% live cells). This lack of cytotoxicity indicates that these MPs could be used in cellular assays in the future.

### Building upon Previous Studies

Typically, carbodiimide chemistry with compounds such as EDC (1-ethyl-3-(3-dimethylaminopropyl)carbodiimide) and NHS (N-hydroxysuccinimide) is used to activate carboxylic acid groups to facilitate amide bond formation with amine groups. However, for our small-scale reaction between silk fibroin and IONPs-GSH, employing these toxic coupling reagents presented challenges for removal after the coupling step. Therefore, we developed an alternative, cleaner conjugation strategy, taking advantage of the silk microsphere salting out method. The pH increase to ∼8 during microsphere formation inherently activated both the silk carboxyl groups and the GSH amine groups by deprotonation, improving their reactivity with each other without needing problematic coupling agents. With thorough mixing during microsphere formation, sufficient contact between the activated groups was expected to enable the formation of amide bonds in the absence of condensing agents. This approach also enabled simultaneous conjugation and nanoparticle trapping within the microspheres as they formed. By circumventing the use of condensing agents, we aimed to simplify the approach, reduce chemical complications, and optimize the biocompatibility of the material for its intended application. Overall, increasing the pH during microsphere formation allowed straightforward amide bond formation between silk and nanoparticles without requiring carbodiimide or other coupling agents. The size of these particles indicated that addition of IONPs-GSH produced smaller particles. However, as the Coulter Counter relies on movement of the particles, if these SIMPs were denser than nonconjugated SIMPs-N, they could more rapidly sink to the bottom, thus measuring the size of the particles as smaller than they are. The SIMPs could more rapidly sink because the IONPs-GSH would be chemically bonded to the silk, causing both the MPs and the IONPs (which are dense) to sink together. Figure 3, which showed representative images of each MP type, demonstrated a relatively similar size between all samples. In terms of zeta potential, all particles had negative zeta potentials.

Prior studies have rarely confirmed or characterized chemical bonding between silk fibroin and IONPs. In our work, we prioritized evaluating the magnetic responsiveness enabled by conjugation. As will be further discussed in the next section, the demonstrated magnetic separation provided evidence that silk-iron binding was achieved. While more in-depth techniques are needed to directly characterize the bond, the magnetic results supported successful conjugation.

### Comparisons to Other Groups

Various researchers^5,61−63^ have evaluated magnetic properties using techniques such as hysteresis curves. In our study we looked at a practical step of magnetic attraction by evaluating the physical magnetic properties of SIMPs. This separation assay was also performed with the magnet on the side, as the particles could potentially settle due to gravity, while other groups placed their samples directly on top of a magnet for separation.^35,64^ Additionally, introducing SIMPs into a hydrogel provided an additional medium for the particles to move through magnetically.

Other groups have investigated silk fibroin particles for a variety of drug delivery applications. In a previous study by Kucharczyk et al.,^59^ a 2.5-fold increase in the average particle size (from 0.5 to 1.2 μm) was observed as the silk concentration increased from 0.5 mg/mL to 5 mg/mL. Considering that the silk concentration in our study was 50 mg/mL, a higher average particle size obtained for Silk MPs was expected. Additionally, the reduction in particle size, especially in SIMPs-N, indicated the impact of IONP incorporation on disrupting silk particle formation, resulting in smaller MP sizes. These size distributions were further confirmed with a Coulter Counter. Additionally, all zeta potentials were negative, which was also seen in Kucharczyk et al.;^5^ their zeta potentials were less negative than those achieved in this study. Seib et al.^65^ investigated drug release from silk only nanoparticles. The silk nanoparticles alone showed low cytotoxicity (<10%) in MCF-7 cells, similar to our Silk MPs and SIMPs. Only when doxorubicin was added did the cytotoxicity increase, indicating the cargo was cytotoxic and not silk particles. Bessa et al.^24^ used MPs to encapsulate BMP-2, BMP-9, or BMP-14. Even though their MPs were fabricated using ethanol precipitation, these MPs were 2.7 ± 0.3 μm in diameter, a size similar to that of our Silk MPs and SIMPs. Outside of silk MPs, magnetic particles with other biomaterials have also been utilized for localized and targeted treatment. Bi et al.^66^ chemically conjugated urokinase to magnetic nanoparticles to induce targeted clot breakdown.

### Limitations

While our EDS characterization results in Figures 3g and S2 showed that IONPs were colocated around or on the SIMPs, more work is needed to spatially map the distribution of the IONPs throughout the particle volume. EDS analysis confirmed the presence of iron at the particles, but it did not distinguish between iron on the inside or outside of the SIMPs. Combining cross-sectional microscopy with elemental mapping, while challenging, could provide more accurate spatial mapping of iron if needed in the future for comparing SIMPs and SIMPs-N. Importantly, our results on separation and magnetically directed mobility are promising and indicate successful anchoring of IONPs to the Silk MPs.

The particle sizes of as-fabricated SIMPs generally varied from submicrometer to a few micrometers in diameter. This variation in particle sizes was most likely caused by the high concentration of RSF used, as shown in the literature.^58^ If a more monodisperse size distribution of SIMPs is required for some application, particles could be sorted after fabrication, as in the work by Li et al.,^67^ in which small particles were precisely sorted based on size using an acoustofluidic device.

Additionally, due to the high concentration of IONPs used for SIMP fabrication, any excess iron was likely to segregate or aggregate. Continuous proper mixing and agitation throughout the salting out process can aid in reducing IONP aggregation. To further reduce this aggregation and segregation of IONPs, the conjugation process efficiency could be enhanced to increase the percentage of IONPs chemically bonded to the silk fibroin rather than existing as unbound nanoparticles prone to aggregation.

While the separation assay showed successful magnetic movement across various concentrations of IONPs, increasing the IONP concentration past 20 mg/mL also showed some interference with the calculated protein concentration from the Lowry assay (Figure S8). As the Lowry assay is based on light absorbance, the interference could be attributed to the high concentration of dark IONPs. Thus, some of the increase in silk protein concentration between magnetic and non-magnetic fractions at higher IONP concentration may be attributed to the iron interference. However, the SEM images still show promise that the SIMPs had higher concentrations of MPs in their magnetic fraction compared to their non-magnetic fraction. These images were qualitative and depend on where in the field of view the image was taken. Magnetic movement through the fibrin gel was performed with only one batch of Silk MPs and SIMPs. This magnetic movement through the gel was also performed as the gel was polymerizing, not following the polymerization reaction. Thus, the viscosity of the hydrogel was potentially closer to that of solution rather than a fully gelled hydrogel. Since the Prussian blue only stains for iron, the IONPs may have been removed from the SIMPs, making it difficult to differentiate between SIMPs and large IONP aggregates. However, our Lowry assay data and SEM images both indicated that SIMPs can be moved magnetically in solution. Additionally, due to the sedimentation of the heavy IONPs, the MPs had to be resuspended frequently. The equal distribution of MPs across each replicate could have been variable.

Cytotoxicity was also only evaluated for 24 h and at this singular time point, which does not capture how cytotoxic the particles would be over time.^68^ Other assays such as MTT could analyze the cellular activity of the entire well,^68^ as opposed to being biased to a specific area of the well for analysis.

### Clinical Applications

Magnetic properties such as magnetic guidance and magnetic responsivity are desirable characteristics for clinical drug delivery systems. One *in vitro* study used an alternating magnetic field to move magnetic silk fibroin particles deeper into the skin.^1^ The alternating magnetic field mixed with a stationary magnetic field allowed for deeper skin penetration than nonmagnet exposed controls. Another group^9^ used an external magnetic field to induce hyperthermia for tumor eradication in a mouse model. Specifically, their magnetic particle imaging technology allowed for hyperthermia to be effective and localized to the tumor of interest, with negligible damage to surrounding organs. This study demonstrated that magnetic particles can be localized to a site of interest and reduce the off-target effects of the treatment. Loading SIMPs with a drug of interest could have a similar outcome as they were also magnetically moved. Magnetic particles are also a strong draw for cancer treatment. An *in vivo* study involving magnetic localization showed that systemic delivery of silk iron nanoparticles could be localized to a tumor in a mouse model.^1,34^ In their study, they used fluorescent imaging to track where their doxorubicin loaded silk iron particles accumulated; magnetically localized particles were more concentrated at the tumor site. The versatility of magnetic particles has clear clinical applications from theranostics to drug delivery.

## Conclusion

GSH conjugation to IONPs and the successful integration of IONPs-GSH with silk fibroin were achieved. A consistent secondary structure was shown across various batches, highlighting reproducibility, and the smoother surface as well as increased β-sheet content for SIMPs indicates improved interactions between IONPs-GSH and silk fibroin. We demonstrated stability for long-term storage, biodegradability once exposed to an enzyme, and noncytotoxicity, as well as magnetic movability through both solution and hydrogel, indicating that they are localizable. Based on this study, we conclude that SIMPs could potentially be loaded with bioactive cargo that can be magnetically localized and directed for treatment of a variety of diseases.
