# Supplementary material for: Chemical Conjugation of Iron Oxide Nanoparticles for the Development of Magnetically Directable Silk Particles
Source: ACS Appl Mater Interfaces. 2025 Feb 3;17(6):8901–13. doi: 10.1021/acsami.4c17536 (PMC11826889; doi:10.1021/acsami.4c17536)
Supplement: Supplementary file 1 — am4c17536_si_001.pdf [file am4c17536_si_001.pdf]

Supporting Information

Chemical Conjugation of Iron Oxide Nanoparticles for the Development of Magnetically Directable Silk Particles

Ande X. Marini[†,#], *Golnaz N. Tomaraei*[‡,#], *Justin S. Weinbaum*[†,§,∥], *Mostafa Bedewy*[⊥,‡,6,^], *and David A. Vorp,*[*,†,§,⊥,6,7,8,9,10,^]

# and ^ indicate equal contribution. #A.X.M. and #G.N.T. contributed equally to this manuscript and ^M.B. and ^D.A.V. contributed equally for supervising authorship to this manuscript.

*indicates corresponding author

[†]Department of Bioengineering, University of Pittsburgh, Pittsburgh, PA, 15261, United States

[‡]Department of Industrial Engineering, University of Pittsburgh, PA, 15261, United States

[§]McGowan Institute for Regenerative Medicine, University of Pittsburgh, Pittsburgh, PA, 15219, United States

[∥]Department of Pathology, University of Pittsburgh, Pittsburgh, PA, 15261, United States

[⊥]Department of Mechanical Engineering and Materials Science, University of Pittsburgh, PA, 15261, United States

[6]Department of Chemical and Petroleum Engineering, University of Pittsburgh, PA, 15261, United States

[7]Department of Surgery, University of Pittsburgh, Pittsburgh, PA, 15213, United States

[8]Department of Cardiothoracic Surgery, University of Pittsburgh, Pittsburgh, PA, 15213, United States

[9]Clinical & Translational Sciences Institute, University of Pittsburgh, Pittsburgh, PA, 15261, United States

[10]Magee Women's Research Institute, University of Pittsburgh, Pittsburgh, PA, 15213, United States

Address Correspondence:

David A. Vorp, Ph.D.

John A. Swanson Professor of Bioengineering

Professor of Cardiothoracic Surgery, Surgery, Chemical and Petroleum Engineering, Mechanical Engineering and Material Science, and the Clinical and Translational Sciences Institute

University of Pittsburgh

300 Technology Drive

Suite 300, Center for Bioengineering

Pittsburgh, PA 15219

Phone: 412-624-5317

FAX: 412-383-8788 (shared)

e-mail: vorp@pitt.edu

KEYWORDS: Silk microparticles, iron oxide nanoparticles, magnetic targeting, materials characterization, regenerated silk fibroin, magnetic guidance

METHODS

Generation of silk and fabrication of IONPs

2.1.1 Synthesis of RSF solution

Silk cocoons were degummed in 0.05 M sodium carbonate, washed, and air-dried. About 12.5 g of fibroin was dissolved in 9.6 M lithium bromide at 65 °C for 3.5 hours. The solution was dialyzed with periodic water changes over three days to remove lithium bromide. The RSF solution was filtered through cleanroom wipes (Contec™ AMSI0001) and stored at 4 °C. It was used within three days to maintain quality. RSF concentration, ranging from 54 mg/ml to 71 mg/ml, was determined by drying a known volume on a glass slide and re-weighing.

2.1.2 Synthesis of IONPs

In a round-bottom flask under argon (Ar) flow, a solution of 1 g (0.05 molar) $FeCl_2.4H_2O$ and 2.7 g (0.1 molar) $FeCl_3.6H_2O$ in 100 mL ultrapure water was prepared and stirred for 30 minutes. Subsequently, 100 mL of ammonium hydroxide was added dropwise while stirring at 550 rpm. The solution was stirred at room temperature for 30 minutes, then heated to 70 °C for 30 minutes,

and cooled under Ar flow. A neodymium disc magnet (D4020, surface field ~0.53 T, DIYMAG) was used to confine the magnetic nanoparticles during washing with ultrapure water and ethanol. The nanoparticles were dried overnight in a vacuum oven at 45 °C and stored in a desiccator.

2.1.3 Conjugation of IONPs with GSH

Approximately 16 mg of IONPs was added to 480 µL ultrapure water and 160 µL methanol and sonicated for 20 minutes to uniformly disperse the IONPs. Subsequently, 13 mg of GSH was added and the mixture was sonicated for 2.5 hours with periodic agitation of the vial to ensure uniform sonication. A neodymium magnet (D4020, surface field ~0.53 T, DIYMAG), confined the magnetic nanoparticles during sequential washes with ultrapure water and methanol. The nanoparticles were dried overnight in a vacuum oven at 45 °C and stored in a desiccator.

Incorporation of IONPs into RSF solution

For an example of how the RSF solution was diluted, 0.794 ml of a 62 mg/ml RSF solution was mixed with 0.206 ml of ultrapure water. Before dilution, 25 mg of IONPs-GSH or IONPs was sonicated in the required amount of ultrapure water for 20 minutes, then mixed with RSF solution and sonicated for uniform dispersion. The suspensions were frequently agitated until used in microparticle synthesis, as detailed in subsequent sections.

Fabrication of microparticles (MPs)

Following incubation, MPs were centrifuged at 10,000 x g and 4°C for 20 minutes to pellet the particles. The supernatant was removed, and the MPs were resuspended in 1000 µL of ultrapure water. The MPs were subsequently washed 5 times by centrifuging at the same speed and resuspending in the same volume of ultrapure water.

IONP characterization

where were dispersed in methanol and drop-cast onto silicon (Si) chips for SEM using a Zeiss SIGMA 500 VP instrument to assess particle size, shape, and morphology variations.

Thermal gravimetric analysis (TGA) was performed on approximately 4 mg samples of IONPs-GSH and IONPs using a Perkin Elmer STA 6000. Samples were heated from 75 °C to 850 °C at 10 °C/min, and mass loss profiles were recorded to evaluate GSH conjugation efficiency.

X-ray photoelectron spectroscopy (XPS) was utilized to confirm Fe_3O_4 synthesis and examine the elemental composition and chemical states of the nanoparticles. The analysis was conducted with an ESCALAB 250Xi instrument using monochromatic Al Kα X-rays and an X-ray spot size of 250 µm.

FTIR analysis was performed on GSH, IONPs, and IONPs-GSH using a Thermo Nicolet IS50 FTIR spectrometer. Samples were prepared as KBr pellets and analyzed in the 4000-400 cm^{-1} spectral range with 128 scans and a nominal resolution of 4 cm^{-1} to identify functional groups and chemical bonds.

Silk microparticle materials characterization

For SEM, 25 µl of each suspension was cast onto Si wafer chips, air-dried overnight, and mounted on SEM stubs. A Zeiss SIGMA 500 VP microscope was used to examine morphology, with multiple randomly selected areas imaged for each sample. Particle sizes were measured with ImageJ software to obtain average values and standard deviations. EDS analysis was conducted using the same microscope with a voltage > 10 kV, and an 8.5 mm working distance to determine elemental composition.

For FTIR, 35 µl of each suspension was deposited on the attenuated total reflection (ATR) diamond crystal and dried. FTIR spectra were recorded using a Thermo Nicolet IS50 FTIR spectrometer operating in ATR mode with 128 scans at 4 cm^{-1} resolution in the range of 4000-400

cm^{-1}. The Amide I region of the spectra was analyzed to determine variations in the secondary structure of silk fibroin.

Degradation of MPs

To assess the degradation of Silk MPs and SIMPs, 44 µL aliquots were incubated in PBS or mixed 1:1 with Protease XIV (1U/mL, P5147, Millipore Sigma, St. Louis, MO) at 37°C for 1, 4, or 7 days. Samples were centrifuged at 10,000 x g and 4°C for 20 minutes, and the supernatant containing potentially degraded protein was removed. The samples were resuspended in equal volume of ultrapure water. To minimize additional degradation from residual protease, the resuspended samples were promptly cast onto Si wafer chips for SEM, EDS, and FTIR analysis as described previously.

Magnetic moveability analysis with a Lowry assay for protein detection

Two different magnets were used for the different separation assays (protein detection via Lowry assay and physical separation via SEM). The aliquot was placed next to a magnet (S2, surface field ~0.88T, K&J Magnetics Inc, Jamison, PA) for 3 minutes to attract magnetic MPs to the edge of the tube. The non-magnetic fraction was removed and placed in a separate tube. An equal volume of ultrapure water was added to the original tube to resuspend the magnetic fraction. Then 15 µL of original unseparated MPs, magnetic fraction, or non-magnetic fraction was diluted 1:10 into 135 µL of ultrapure water for detection in a Lowry assay (23240, ThermoFisher Scientific). For a larger magnet surface for separation of magnetic and non-magnetic fractions for SEM, a larger neodymium magnet (D93, surface field ~0.44T, K&J Magnetics Inc.) was used. SEM was performed on aliquots of 25 µL of these separated fractions which were drop cast onto Si chips.

For Lowry assays, 40 µL of each sample or a standard curve (in triplicate) of bovine serum albumin (BSA, 0 to 1500 µg/mL) diluted in ultrapure water was added to 96-well plates and mixed

with 200 µL of Lowry reagent. Samples were incubated at room temperature for 10 minutes. Then 20 µL of a phenol reagent was added to each well and incubated for another 30 minutes at room temperature. The plate was read at 750 nm.

Interference analysis of GSH, IONPs, and IONPs-GSH on protein concentration readings

To calculate the amount of interference seen with the IONPs-GSH, varying concentrations (0-50 mg/mL in 10 mg/mL increments) were used with the same standard curve procedure. The slopes of these standard curve comparisons were plotted to see the relationship between IONPs-GSH concentration and interference on protein readout.

Magnetic moveability analysis through a hydrogel

Bovine fibrinogen type 1 (1.2 mL, 3.7 mg/mL, #8630, Millipore Sigma) and 0.21 U/mL bovine thrombin (300 µL, #T7513, Millipore Sigma) were mixed with 300 µL of Silk MPs or SIMPs resuspended in DMEM + Glutamax media (10564-011, Gibco, Gaithersburg, MD). To create the hydrogel, 200 µL of the gel mixture was added to heat-stamped circular molds created with 7.94 mm (5/16") diameter cork borers imprinted in 24 well plates. For groups exposed to the magnet, neodymium magnets (D93, surface field ~0.44T, K&J Magnetics Inc.) were placed next to the gels during the gelation process (both for 10 minutes at room temperature and 45 minutes at 37°C). Control groups without magnetic exposure were incubated for the same period of time. Gels were stored in PBS at 4°C prior to staining.

Fibrin gels were fixed using 4% paraformaldehyde (ThermoFisher Scientific) followed by 3 PBS washes. The gels were stained for 20 minutes at room temperature with a Prussian Blue stain (LC190601, LabChem Inc., Zelienople, PA) mixed 1:1 with a 20% concentrated hydrochloric acid solution. Excess stain was removed with 3 ultrapure water washes. Fibrin gels were imaged using an Olympus ZX7 microscope (Olympus, Lake Success, NY).

Cytotoxicity of MPs

Media was removed from cells and washed with 1 mL of Hank's Balanced Salt Solution (HBSS) (Gibco). Filtered ultrapure water (200 μL) was added to one group of wells for 15 minutes to induce hypotonic lysis of the cells (positive control for cell death). The LIVE/DEAD stain was made according to the manufacturer's protocol by mixing the green LIVE stain 1:1 with the red DEAD stain; this solution was then mixed 1:1 with HBSS for the working solution. Then 180 μL of LIVE/DEAD stain was added to each well and incubated for 20 minutes.

The percentage of live and dead cells was quantified using ImageJ (National Institutes of Health, Bethesda, MD). Images were split into red and green channels and the number of cells were counted in each channel using the ImageJ thresholding function. An optimal threshold for green and red cell counts was determined and used for counting the number of cells in all images. The percentage of live cells was calculated by dividing the number of green cells by the number of green and red cells (total cells). Conversely, the percentage of dead cells was calculated by dividing the number of red cells by the total number of cells.

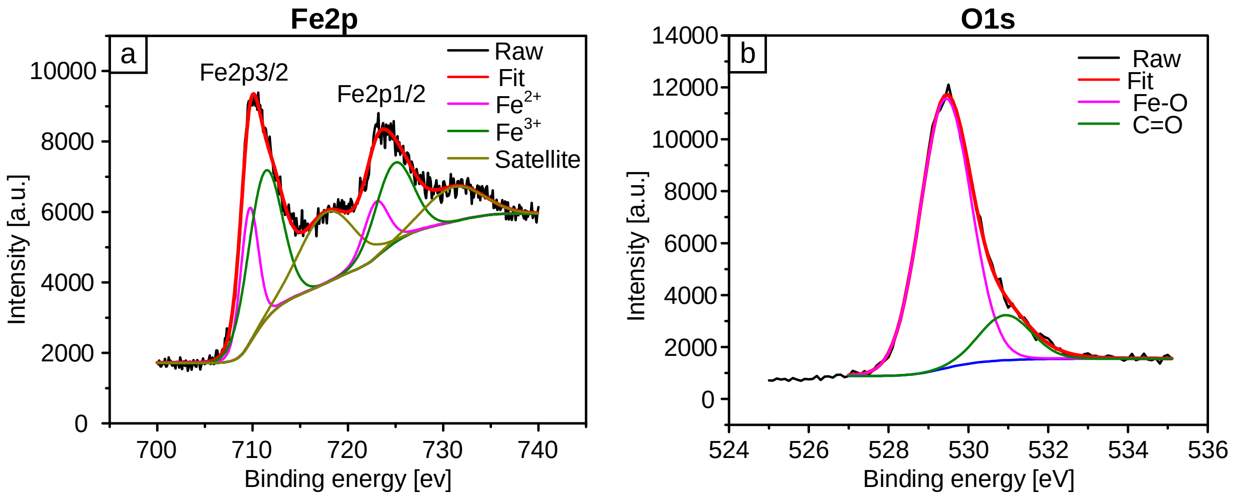

Figure S1. The deconvoluted XPS spectra of (a) Fe2p and (b) O1s for IONPs. In Fe2p spectrum, peaks corresponding to Fe2+ and Fe3+ species, as well as satellite peaks, are labeled. In O1s spectrum, a peak corresponding to O2- ions in Fe3O4 and a peak corresponding to C=O bonds are identified.

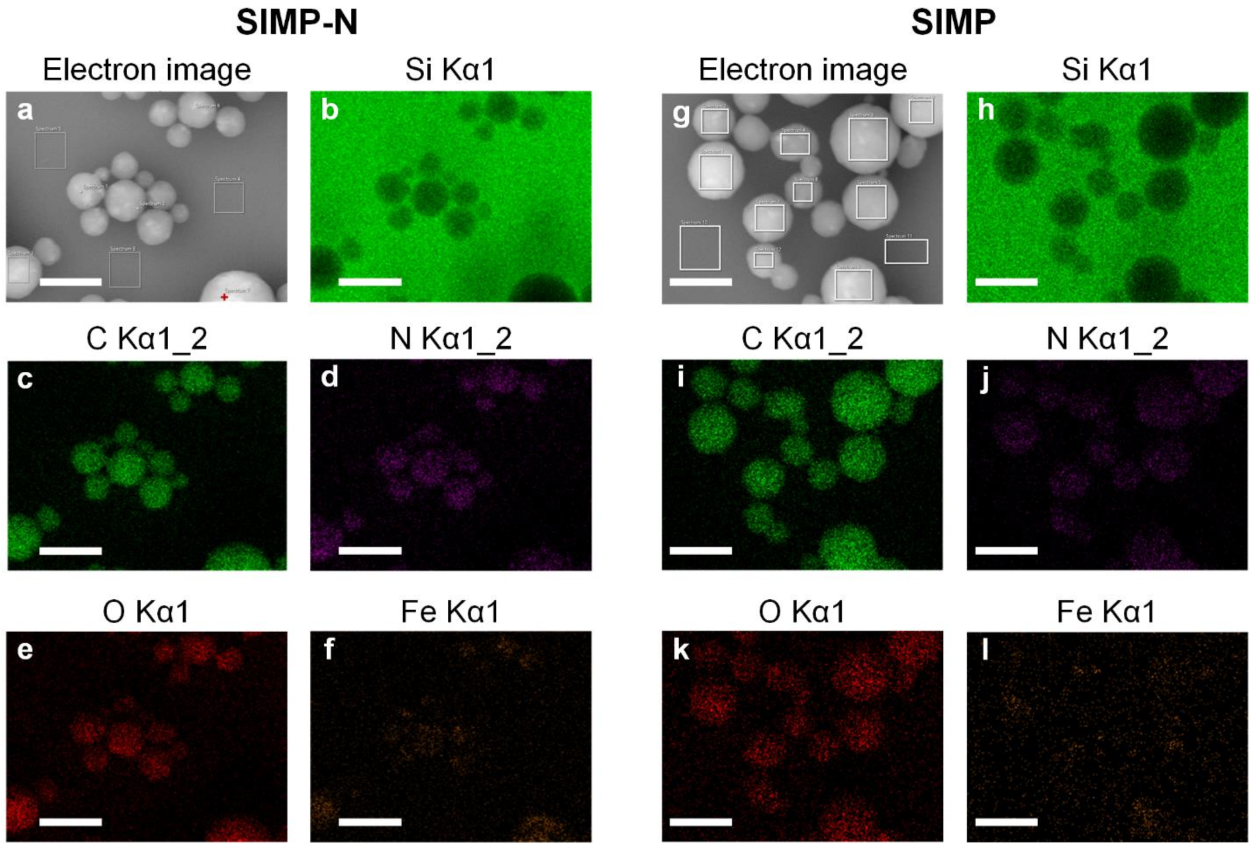

Figure S2. EDS analysis of SIMPs-N (a-f) and SIMP (g-l). Red symbols on electron images (a, g) indicate locations of EDS spectra acquisition from particles and silicon chip substrate. Elemental maps show the spatial distribution of Si (b, h), C (c, i), N (d, j), O (e, k), and Fe (f, l). The C, N, and O maps correlate with particle morphology. However, Fe does not correlate well due to lower content and weaker signal. The scale bars in all panels are 2.5 μm.

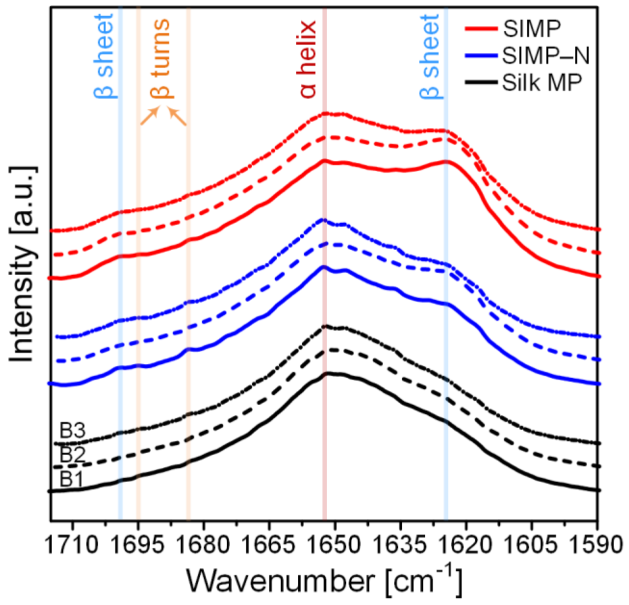

Figure S3. Amide I FTIR spectra of Silk MPs, SIMPs, and Silk-IONP MPs (SIMPs-N) (B1, B2, B3 batches). Consistency observed among batches. Increased β-sheet and β-turn intensity is observed in SIMPs and SIMPs-N with IONPs.

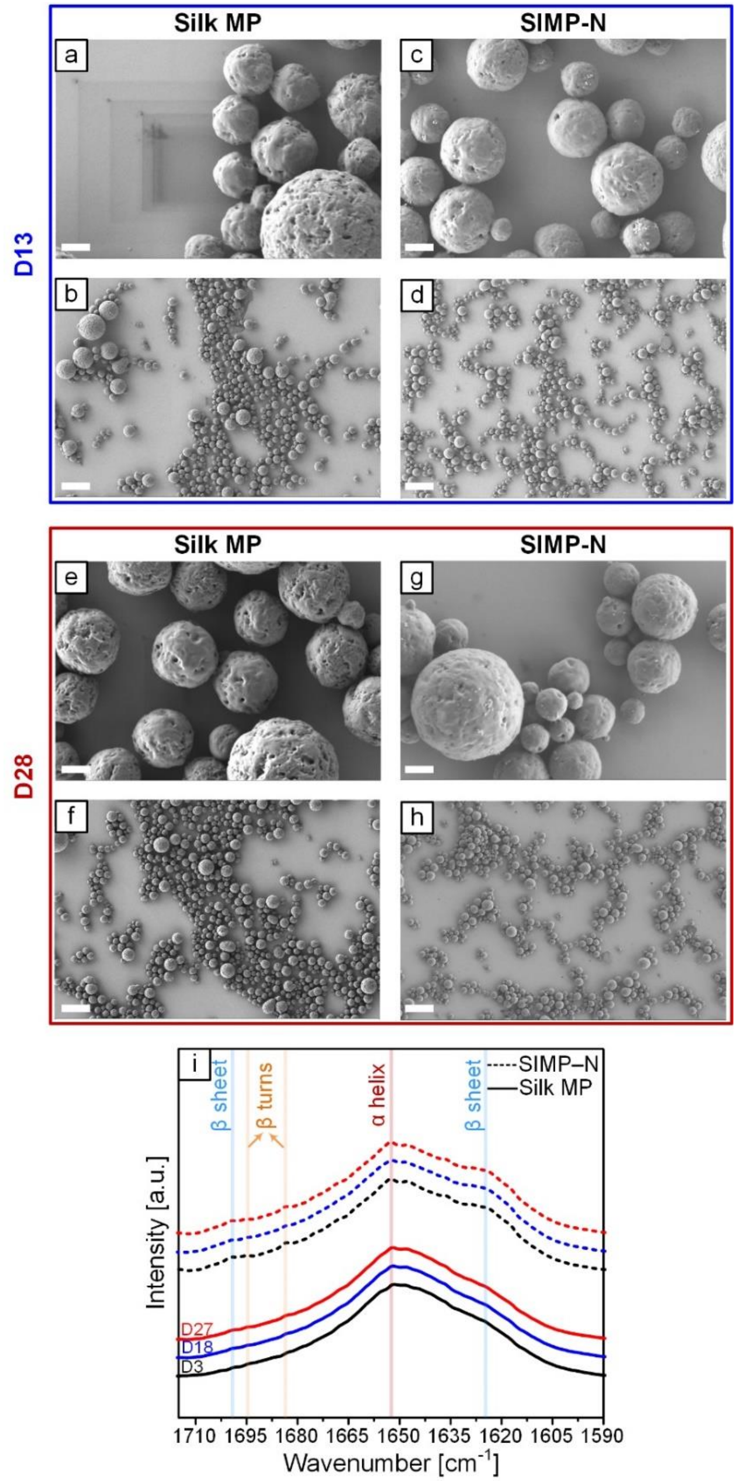

Figure S4. Stability assessment of Silk MPs and SIMPs-N stored at 4°C in the refrigerator for 13 and 28 days. SEM images at D13, at high magnification (a: Silk MPs; c: SIMPs-N) and low

magnification (b: Silk MPs; d: SIMPs-N), and at D28, at high magnification (e: Silk MPs; g: SIMPs-N) and low magnification (f: Silk MPs; h: SIMPs-N), further confirm stability. (i) Amide I FTIR spectra for one batch of Silk MPs and SIMPs-N at D3, D18, and D27 show stable secondary structure. Scale bars: Panels a, c, e, g (1 μm) and panels b, d, f, h (10 μm).

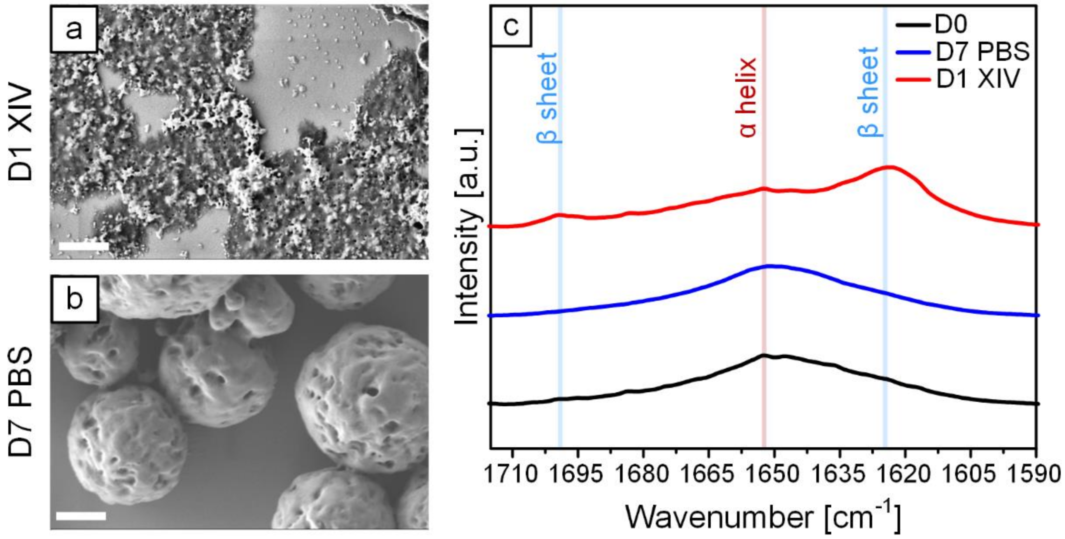

Figure S5. Degradation analysis of Silk MPs incubated in Protease XIV and PBS. SEM image at (a) D1 of incubation in Protease XIV shows signs of degradation, while SEM image at (b) D7 of incubation in PBS show that silk MPs maintain structural integrity and spherical shape. All the scale bars are 1 μm.(c) Amide I FTIR spectra for Silk MPs without incubation (D0), at D1 in Protease XIV, and at D7 in PBS indicate minimal changes over 7 days in PBS, while 1 day in Protease XIV induces substantial β-sheet increase and α-helix reduction, consistent with SEM degradation observations.

SIMPs_ D1 XIV

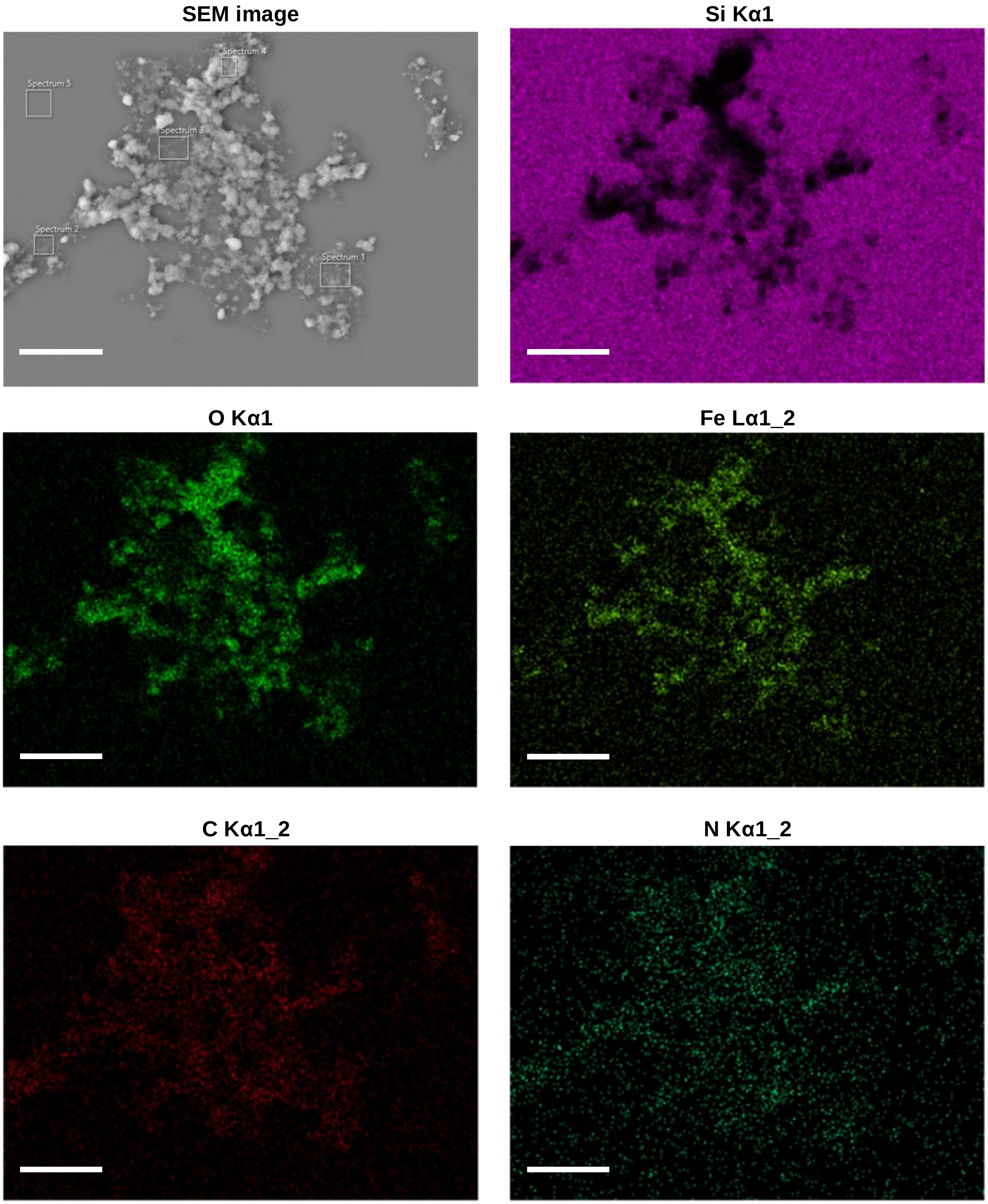

SEM image
Si Kα1
Spectrum 4
Spectrum 5
Spectrum 3
Spectrum 2
Spectrum 1
O Kα1
Fe Lα1_2
C Kα1_2
N Kα1_2

Figure S6. EDS analysis of degraded SIMPs after D1 of incubation in Protease XIV. Elemental mapping results from EDS confirm the primary localization of IONPs within the microparticles and their association with degraded protein. The scale bars in all panels are 10 μm.

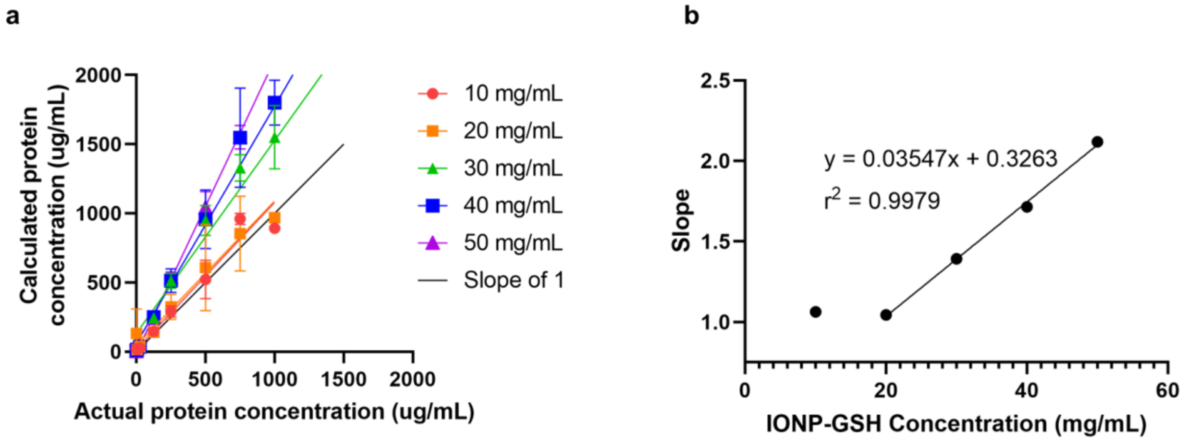

Figure S7. IONPs-GSH interfered with protein concentration measurements obtained using a Lowry assay. When the concentration of IONPs-GSH was at least 20 mg/mL, the measured concentration of protein also increased. A linear comparison between actual BSA protein concentration and detected BSA protein concentration demonstrated a steeper slope as IONP-GSH concentration increased (a). This interference relationship was shown to be linear and calculated by plotting the slope of detected protein/actual protein vs. concentration of IONP-GSH (b).

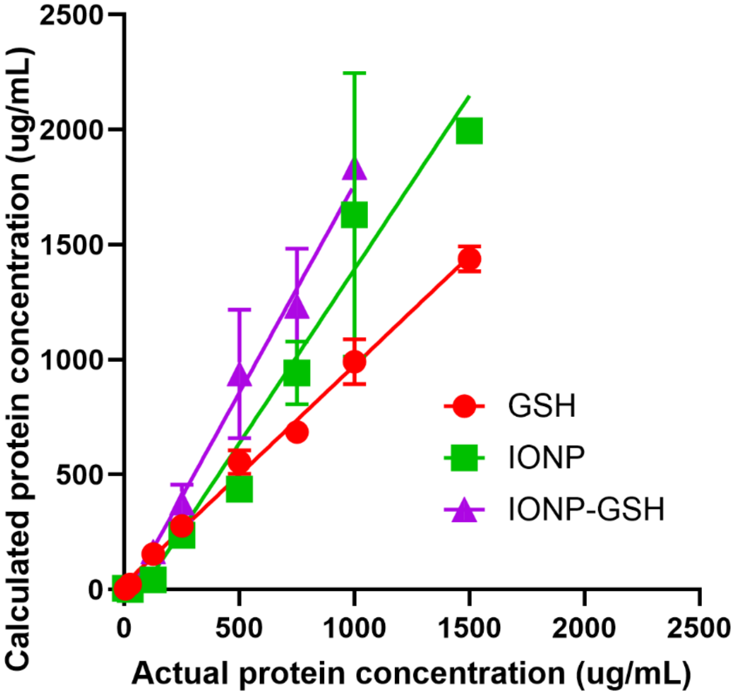

Figure S8. Interference with increasing IONP concentration can be seen while GSH concentration does not affect measured protein concentration. Concentrations of GSH, IONPs, and IONPs-GSH were used to test their inference with measured protein. Mainly IONP concentration affected the measured protein concentration.

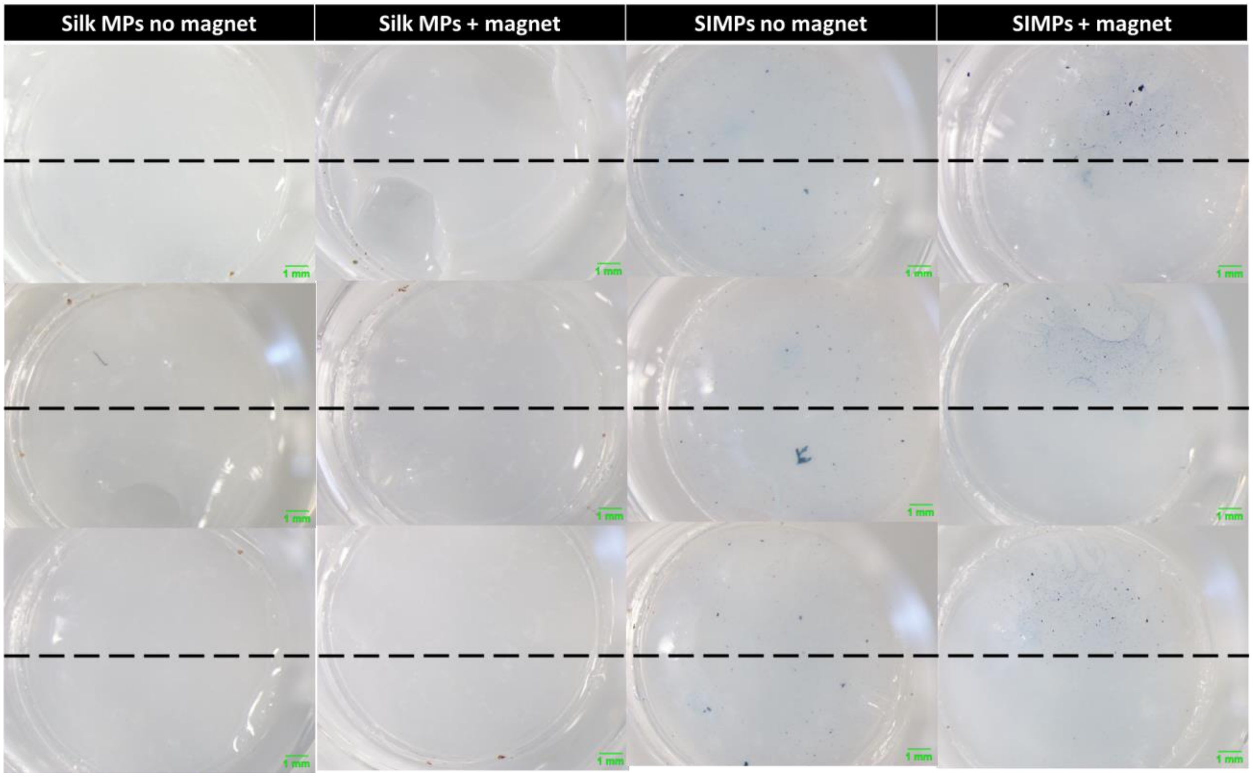

Figure S9. All three images of the SIMPs + magnet fibrin gels demonstrate magnetic localization. Between the different images for each group, it is shown that SIMPs are stained blue compared to Silk MP controls. Additionally, SIMP groups exposed to the magnet show Prussian Blue stained SIMPs are moved toward the top of the gel, where the magnet was located.

Table S1. Fitting parameters for the deconvolution of Fe2p spectrum of IONPs using the XPSPEAK41 software.

Peak position (eV)	Area	FWHM (eV)	% Lorentzian-Gaussian (0: G, 100: L)
709.7	9503.199	2.2	11
711.3	19006.4	4.2	0
717.7	14236.99	6.6	0
722.9	5591.65	3.1	19
724.9	11183.3	4.6	0
731.1	7271.206	6.9	0
